# Cannabidiol-Loaded Solid Lipid Nanoparticles Ameliorate the Inhibition of Proinflammatory Cytokines and Free Radicals in an In Vitro Inflammation-Induced Cell Model

**DOI:** 10.3390/ijms25094744

**Published:** 2024-04-26

**Authors:** Khent Primo Alcantara, John Wilfred T. Malabanan, Nonthaneth Nalinratana, Worathat Thitikornpong, Pornchai Rojsitthisak, Pranee Rojsitthisak

**Affiliations:** 1 Center of Excellence in Natural Products for Ageing and Chronic Diseases, Chulalongkorn University, Bangkok 10330, Thailand; khentprimo.a@chula.ac.th (K.P.A.); jwtmalabanan@gmail.com (J.W.T.M.); nonthaneth.n@pharm.chula.ac.th (N.N.); worathat.t@pharm.chula.ac.th (W.T.); pranee.l@chula.ac.th (P.R.); 2 Department of Food and Pharmaceutical Chemistry, Faculty of Pharmaceutical Sciences, Chulalongkorn University, Bangkok 10330, Thailand; 3 Department of Pharmacology and Physiology, Faculty of Pharmaceutical Sciences, Chulalongkorn University, Bangkok 10330, Thailand; 4 Metallurgy and Materials Science Research Institute, Chulalongkorn University, Bangkok 10330, Thailand

**Keywords:** cannabidiol, solid lipid nanoparticle, drug delivery, inflammation, response surface methodology, Box–Behnken

## Abstract

Cannabidiol (CBD) is a non-psychoactive compound derived from *Cannabis sativa*. It has demonstrated promising effects in combating inflammation and holds potential as a treatment for the progression of chronic inflammation. However, the clinical application of CBD is limited due to its poor solubility and bioavailability. This study introduces an effective method for preparing CBD-loaded solid lipid nanoparticles (CBD-SLNs) using a combination of low-energy hot homogenization and ultrasonication. We enhanced this process by employing statistical optimization with response surface methodology (RSM). The optimized CBD-SLN formulation utilizes glyceryl monostearate as the primary lipid component of the nanocarrier. The CBD-SLN formulation is screened as a potential tool for managing chronic inflammation. Stable, uniformly dispersed spherical nanoparticles with a size of 123 nm, a surface charge of −32.1 mV, an encapsulation efficiency of 95.16%, and a drug loading of 2.36% were obtained. The CBD-SLNs exhibited sustained release properties, ensuring prolonged and controlled CBD delivery, which could potentially amplify its therapeutic effects. Additionally, we observed that CBD-SLNs significantly reduced both reactive oxygen and nitrogen species and proinflammatory cytokines in chondrocyte and macrophage cell lines, with these inhibitory effects being more pronounced than those of free CBD. In conclusion, CBD-SLNs demonstrated superiority over free CBD, highlighting its potential as an effective delivery system for CBD.

## 1. Introduction

Cannabidiol (CBD) is a non-psychoactive cannabinoid derived from *Cannabis sativa*. Unlike tetrahydrocannabinol, which directly binds to cannabinoid receptors CB1 and CB2, CBD exerts its effects through allosteric modulation of these receptors [1,2]. This unique mechanism of action has been linked to various pharmacological effects that show promise for clinical applications. Studies indicate its potential as an anticancer agent, effectiveness in epilepsy and seizures, and prospective benefits for neurodegenerative and cardiac conditions [3]. CBD’s notable feature is its ability to exhibit anti-inflammatory and antioxidant effects by activating peroxisome proliferator-activated receptors (PPARs) directly or indirectly [4]. Clinical research has shown that CBD can lower the levels of proinflammatory cytokines, inhibit T cell proliferation, promote T cell apoptosis, and reduce the migration and adhesion of immune cells [3].

Chronic inflammation has emerged as a critical contributor to a spectrum of disorders affecting millions globally [5]. These inflammation-related conditions intricately involve multiple organs and systems, resulting in pain, tissue damage, and compromised functionality. Prominent examples include autoimmune diseases, exemplified by rheumatoid arthritis; cardiovascular diseases, like atherosclerosis; and musculoskeletal disorders, such as osteoarthritis and gout [6]. The excessive secretion of major proinflammatory cytokines, specifically IL-1, IL-6, and TNF-α, play crucial roles in disrupting metabolism and exacerbating catabolic processes within the pathophysiology of inflammatory disorders [7]. A promising avenue for intervention is the use of CBD, which has been shown to elevate intracellular calcium levels, reduce cell viability, and decrease IL-6, IL-8, TNF-α, IL-17A, and matrix metalloproteinase-3 (MMP-3) production in rheumatoid arthritis synovial fibroblasts (RASFs) and other inflammatory diseases including psoriasis [8,9]. Understanding these intricate cellular processes is imperative for developing interventions that have the potential to slow down the progression of pervasive chronic inflammatory conditions.

However, CBD is classified under the Biopharmaceutics Classification System as a class II drug, characterized by high lipophilicity (logP = ~6.97), pKa of 10.6, and low water solubility (28.0 mg/L) [10,11]. These physicochemical properties lead to the poor, unpredictable, and inconsistent absorption patterns of cannabinoids. The oral bioavailability of CBD in humans is also limited, typically ranging from 9 to 13%, attributed to its low water solubility and significant degradation during the first pass metabolism [12]. Cytochrome P450 (CYP) enzymes, notably CYP3A4 and CYP2C19, metabolize CBD into its 7-OH derivative, losing approximately 75% of the drug that enters the systemic circulation [1,12]. Numerous nanotechnology-based formulation strategies have been developed to improve the delivery of cannabinoids, address their inherent limitations, and enhance their biopharmaceutical properties. One approach involves encapsulating cannabinoids within nanocarriers, protecting the core cannabinoid compounds from degradation, augmenting their physicochemical stability, and increasing their bioavailability. It was previously introduced that a polymeric nanoparticle-based delivery system for CBD has been developed to enhance bioavailability and therapeutic efficacy in treating chronic inflammation, precisely osteoarthritis [13]. Despite extensive research in nanotechnology, previous studies have not specifically addressed the formulation of CBD into lipid-based nanoparticles, incorporating statistical optimization in the process and screening for the formulation’s efficacy in managing chronic inflammatory disorders in a chondrocyte cell model.

Lipid-based nanoparticles have gained significant attention for their ability to encapsulate hydrophobic active compounds effectively, making them a versatile choice for various applications. Several studies have focused on encapsulating CBD in lipid nanoparticles, emphasizing its potential in cancer therapy, infectious diseases, and chronic conditions [14,15,16]. These lipid nanoparticles, particularly solid lipid nanoparticles (SLNs), offer numerous advantages, including cost effectiveness, chemical versatility, and biodegradability. Unlike polymer carriers, SLNs comprise biodegradable lipids, which may facilitate regulatory approval [17]. The size of NPs, including SLNs, is a crucial factor in regulatory approval for medicinal products, as emphasized by Clogston et al. [18]. The advent of nanotechnology-based drug delivery systems has garnered significant attention from regulatory bodies worldwide, notably the Food and Drug Administration (FDA) in the United States and the European Medicines Agency (EMA) in Europe. These agencies rigorously evaluate nanomedicines for safety, efficacy, and quality, paying particular attention to particle size, distribution, and their potential to enhance bioavailability and targeted delivery. Over sixty NP formulations have been approved in the US and EU, while many others are in clinical or preclinical development, indicating a concerted effort to translate promising bench research into commercially viable pharmaceutical products [18]. Recent approvals of several nanoformulations for clinical use emphasized a burgeoning acceptance of nanotechnology within therapeutic contexts [19,20]. Though explicit examples of SLN-based medicinal products currently approved for marketing remain sparse, the successful commercialization of other lipid-based nanoparticles, like liposomes and nanoemulsions, offers a promising outlook for SLNs in overcoming regulatory hurdles [21,22].

SLNs have emerged as a versatile platform for delivering hydrophobic active substances, offering enhanced bioavailability and therapeutic efficacy across various administration routes. For instance, CBD, a hydrophobic compound known for its potential in treating chronic inflammatory conditions, has been effectively incorporated into SLNs. The formulation allows for both oral and oral applications, highlighting the adaptability of SLNs in delivering hydrophobic compounds. Additionally, asiatic acid, an active compound found in *Centella asiatica* L., has been successfully loaded into SLNs to enhance its absorption in the nasal cavity, demonstrating the versatility of SLNs in novel delivery techniques [23]. In dermatological applications, linezolid, a synthetic oxazolidinone antibiotic, has been formulated into solid SLNs for dermal delivery to manage skin and soft tissue infections [24]. This approach allows for localized and sustained release of linezolid at the site of infection, reducing the need for systemic administration and minimizing associated side effects. Similarly, nintedanib, an anti-fibrotic agent used in treating idiopathic pulmonary fibrosis (IPF), has been successfully encapsulated in SLNs to enhance its oral bioavailability and therapeutic efficacy [25]. This formulation strategy aims to reduce the required dosage of NIN, thereby mitigating liver and gastrointestinal toxicities associated with higher doses. These examples collectively highlight the versatility and effectiveness of SLNs in encapsulating hydrophobic compounds, enabling targeted delivery and controlled release for improved therapeutic outcomes while minimizing adverse effects. Recent research explored the potential of using SLNs to deliver tocilizumab-tailored CBD-loaded SLNs, which could be valuable in managing SARS-CoV-2 and related infections by leveraging CBD’s anti-inflammatory properties [15]. Beyond infectious diseases, SLNs have versatile applications in drug delivery addressing neurological disorders and cancer treatment. SLNs were found to improve absorption, reduce toxicity, and combat antibiotic resistance by encapsulating antimicrobial drugs while inhibiting bacterial efflux mechanisms [26]. Moreover, SLNs are promising carriers in the food industry, enhancing products’ quality and nutritional value by protecting delicate compounds like vitamins and minerals during digestion [17]. They are commonly used to fortify food products with essential micronutrients for human health and as delivery agents for health-beneficial components, including medicinal compounds and antioxidants [27].

This study evaluates the therapeutic potential of CBD-SLNs in a controlled environment, aiming to provide preliminary insights into their effectiveness in treating inflammation. While promising, the outcomes of this research serve as a foundation for future investigations to fully explore the potential of CBD-SLNs in clinical applications. CBD-SLNs are designed as a straightforward yet effective delivery system to enhance CBD’s physicochemical properties and biological activity. By encapsulating CBD in SLNs, we aim to improve its chondroprotective effects by targeting key inflammatory factors like TNF-α and IL-6. Here, we provide detailed insights into the pharmacological framework of our CBD-SLNs, emphasizing the established groundwork for further developing the possible route of administration and dosage form. Specifically designed for oral administration, these CBD-SLNs are tailored for local and systemic inflammation typical of chronic inflammatory conditions affecting bones and joints [28]. The formulation of these nanoparticles as an aqueous dispersion is finely optimized to enhance its absorption and facilitate the sustained release of CBD within the body [29]. This strategic delivery approach harnesses the physicochemical properties of SLNs to address the challenges posed by CBD’s hydrophobic nature, aiming to optimize therapeutic efficacy [30]. Understanding the inherent challenges associated with the bioavailability and degradation of CBD, our study has placed a significant emphasis on these aspects during the formulation of our SLNs. CBD, a compound with notable therapeutic potential, faces limitations in clinical applications due to its low bioavailability and susceptibility to degradation [31,32]. We have optimized the encapsulation efficiency (EE) and drug loading (DL) parameters of our SLNs through meticulous formulation strategies to address these challenges. Our approach involves a comprehensive analysis to enhance CBD’s bioavailability and minimize degradation, resulting in an efficient delivery system that maximizes its therapeutic benefits [31,33]. By leveraging CBD’s pharmacological advantages, we aim to extend its applicability in treating chronic conditions, underscoring the importance of advanced drug delivery systems in overcoming the pharmacokinetic limitations associated with hydrophobic compounds. The study employs a quality-by-design (QbD) approach, which examines variables and their interactions through a mathematical model. The response surface method (RSM) with a Box–Behnken design (BBD) determines optimal conditions, leading to a stable CBD-SLN formulation with sustained release and potentially increasing bioavailability. In vitro tests show that the optimized CBD-SLN formulation effectively reduces proinflammatory cytokines and reactive oxygen and nitrogen species (RONS). The promising results of our study could pave the way for developing effective treatments for chronic inflammatory diseases of the bones and joints. While primarily exploratory, this study contributes crucial insights into the formulation and efficacy of CBD-SLNs, laying the groundwork for future therapeutic strategies. The significance of our findings lies in their potential to enhance the bioavailability and therapeutic profile of CBD, a compound of increasing interest for its anti-inflammatory and analgesic properties. Furthermore, our research underscores the imperative for further investigation, particularly through in vivo studies, to validate these initial outcomes and explore the full spectrum of CBD-SLNs’ therapeutic possibilities. We hope this work will catalyze the advancement of SLN-based formulations from laboratory settings to the forefront of clinical practice, offering new avenues for treating chronic inflammatory diseases and beyond.

## 2. Results and Discussions

### 2.1. Response Surface Analysis and Optimization of CBD-SLNs

The CBD-SLN formulation conditions were optimized using a three-factor BBD with Design-Expert^®^ software (Table 1). Three key factors studied were glyceryl monostearate (GMS, A), polysorbate 80 (B), and methanolic CBD (C), aiming to determine their impact on SLN characteristics, including size (Y_1_), polydispersity index (PDI, Y_2_), encapsulation efficiency (EE, Y_3_), and drug loading (DL, Y_4_), as outlined in Table 2. Analysis of variance (ANOVA) was employed to compare the variations arising from changes in factor combinations with random measurement errors. The statistical analysis results are summarized in Supplementary Tables S1–S5.

**Figure 1:**
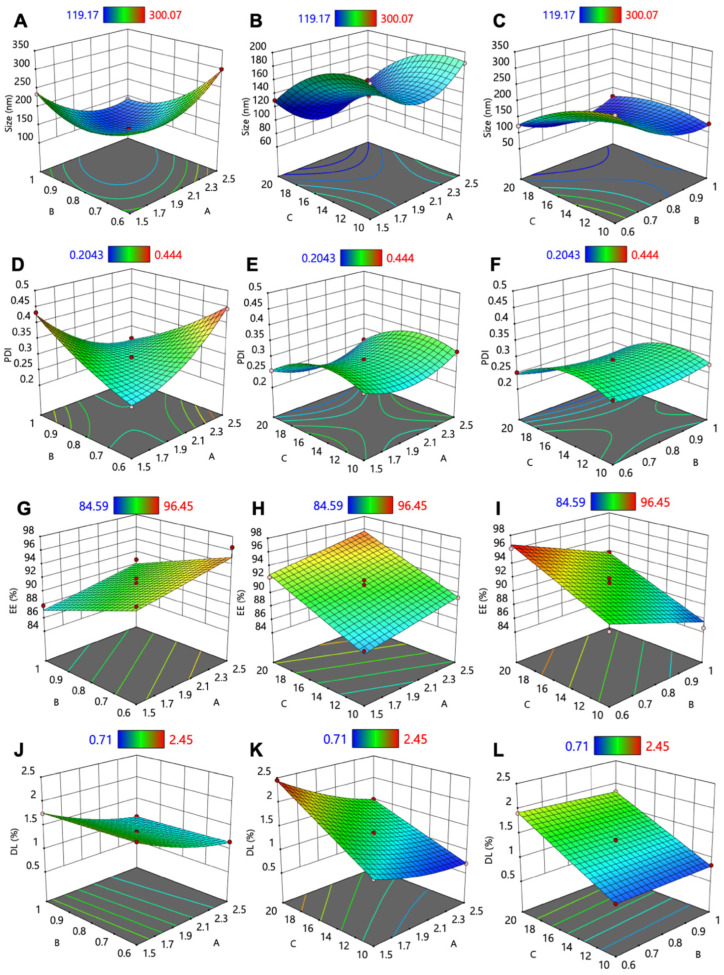
Three-dimensional surface model plots showing the interaction of the independent factors to (**A**–**C**) particle size, (**D**–**F**) PDI, (**G**–**I**) EE, and (**J**–**L**) DL.

The optimal model was chosen based on statistical significance, lack of significant lack-of-fit, high adjusted and predicted R^2^ values, and adequate precision. Regression equations (Equations (1)–(4)) were generated for each response to assess the impact of factors; positive coefficients indicated a positive or synergistic effect, while negative coefficients indicated the opposite. Three-dimensional response surface plots (Figure 1) were utilized to visualize how two factors influenced dependent variables while keeping the third constant. This systematic approach enabled the efficient optimization of CBD-SLNs.

The size of lipid-based nanoparticles is a crucial parameter that profoundly influences numerous factors including stability, EE, drug release kinetics, biodistribution, mucoadhesion, and cellular uptake [34]. For example, it is essential for intra-articular delivery as smaller sizes are more likely to penetrate the cartilage extracellular matrix (ECM) [35]. The size of the CBD-SLN formulations ranged from 119 to 300 nm. The response surface analysis (Figure 1A,B) and the regression equation of the fitted model (Equation (1)) revealed that the surfactant (B) (F-value = 1012.31; *p*-value = <0.0001) was the most significant factor in decreasing the size of the SLNs.

**Particle size (Y_1_)** = +137.91 − 5.26A − 35.20B − 33.95C − 42.65AB + 32.12BC + 43.62A^2^ + 43.15B^2^ − 24.08C^2^ (1)

The PDI of any nanoparticles serves as a measure of their size heterogeneity. In drug delivery applications involving lipid-based carriers, a PDI equal to or below 0.3 is considered an acceptable criterion [34]. In this study, the recorded PDIs ranged from 0.2043 to 0.4444. The response surface analysis (Figure 1D–F) and the regression equation of the fitted model (Equation (2)) revealed that the combined effect of the amount of lipid (A) and surfactant (B) emerged as the primary factor (F-value = 320.76; *p*-value < 0.0001) responsible for decreasing the PDI value of the SLNs. The size reduction, leading to a monodispersed system at higher surfactant concentrations, can be attributed to decreased interfacial tension between the aqueous and lipid phases. This enables the dispersion of tiny emulsion droplets, leading to the formation of smaller SLNs.

Furthermore, the selection of surfactant concentration was carefully optimized to enhance the stability of the particles within the formulation, preventing agglomeration and ensuring their long-term stability. This consideration is pivotal, especially given that our SLNs are designed for oral application [36]. In this study, the oral route was identified as the intended administration path for our CBD-SLN formulation, necessitating an optimized surfactant concentration to balance NP stability with GI physiology compatibility. The selection of surfactants was informed by a thorough literature evaluation of their safety profiles, effectiveness in nanoparticle stabilization, and the possibility of enhancing the absorption of CBD. Such criteria ensured our formulation’s alignment with the existing literature on oral applications [37,38,39].

Moreover, small emulsion droplets serve as SLN precursors, transforming into smaller, uniform nanoparticles during solidification. Monodispersity benefits SLN formulations by ensuring consistent drug nanoparticle distribution, enhancing performance, and therapeutic efficacy. Moreover, the surfactant also enhances storage stability and shelf life by forming a protective layer around CBD-SLNs.

**PDI (Y_2_)** = +0.2888 + 0.0045A − 0.0081B − 0.0261C − 0.0913AB + 0.0432A^2^ + 0.0152B^2^ − 0.0491C^2^ (2)

The drug EE and DL are vital parameters in the field of nanomedicine. The EE signifies the effectiveness of drug utilization during the preparation of nanoparticles, while DL represents the mass ratio of drugs to nanoparticles [40]. The EE obtained from the experiments ranged from 84 to 96%. The response surface analysis (Figure 1G–I) and the regression equation of the fitted model (Equation (3)) revealed that the concentration of the drug was identified as the primary factor (F-value = 106.92; *p*-value < 0.0001) responsible for increasing the EE of the SLNs, likely due to the high lipophilicity of CBD. In contrast, high surfactant concentration reduces EE by increasing CBD’s solubility in water, reducing its affinity for the lipid phase. More surfactants can also lead to CBD solubilization in the aqueous phase, further decreasing EE. [36].

**EE (Y_3_)** = +91.12 + 1.44A − 2.47B + 3.10C (3)

Moreover, the DL obtained from the experiments ranged from 0.7 to 2.4%. The response surface analysis (Figure 1J–L) and the regression equation of the fitted model (Equation (4)) revealed that the CBD concentration (C) (F-value = 11055.40; *p*-value < 0.0001) and the amount of GMS (A) (F-value = 4866.04; *p*-value < 0.0001) were identified as the primary factors responsible for increasing the DL of the SLNs. The EE and DL capacity of SLNs are intricately linked to the composition of the lipid matrix and its crystalline state [41]. Higher drug concentration in the formulation increases drug molecules available for SLN incorporation. More lipids create space, promoting further drug encapsulation due to increased surface area. Consequently, a rise in lipid concentration generally results in an enhanced DL capacity [42]. Therefore, the interplay between drug concentration and lipid content is pivotal in optimizing SLNs for efficient drug delivery applications.

**DL (Y_4_)** = +1.36 − 0.3425A − 0.0338B + 0.5163C − 0.1250AC − 0.0175BC + 0.0979A^2^ (4)

### 2.2. Validation of the Optimized Conditions in Formulating CBD-SLNs

In the development of our CBD-SLN, we determined the optimal formulation conditions to be GMS at 1.60 g, polysorbate 80 at 0.62 g, and CBD at 20 mg. This composition was optimized through systematic testing to enhance SLN characteristics such as particle size, DL, and EE, as detailed in Table 3. The use of methanolic CBD involved using a minimal amount of methanol to dissolve the CBD during the initial formulation stages. This choice was due to methanol’s ability to quickly evaporate at the high temperatures used for lipid melting, effectively leaving no residual solvent in the final product. To confirm the accuracy and precision of the RSM, the CBD-SLNs were prepared under the optimal conditions determined for the quantities of GMS (A), polysorbate 80 (B), and CBD concentration (C), as specified in Table 3. Subsequently, the observed values for size (Y_1_), PDI (Y_2_), EE (Y_3_), and DL (Y_4_) obtained from the experiment were compared with the predicted responses generated by the software. The analysis of the predicted values against the observed values for each response showed a high level of agreement within the 95% confidence interval (Table 3). Additionally, the computed error was found to be <10% (Equation (5)), further indicating the excellent accuracy and precision of the RSM models used in predicting the outcomes of the experiment.

**% Error** = (observed value − predicted value)/predicted value × 100 (5)

### 2.3. Characterization of the CBD-SLNs

**Figure 2:**
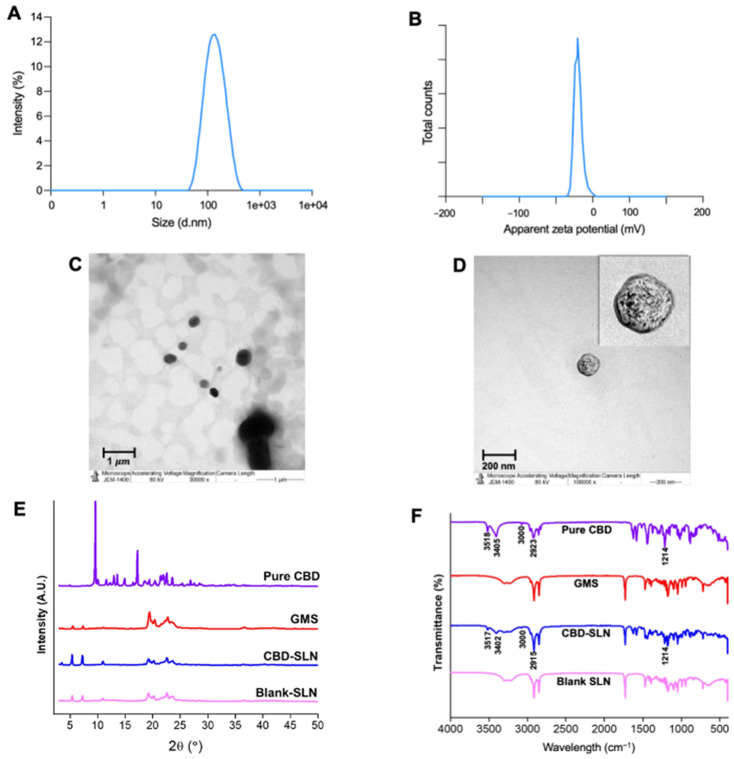
Physicochemical characteristics of the optimized CBD-SLNs. (**A**) Size distribution, (**B**) zeta potential distribution, TEM micrographs at (**C**) 30,000× and (**D**) 100,000× magnifications, (**E**) XRD pattern, and (**F**) FTIR spectra of CBD-SLNs, blank SLNs, and their components.

The optimal formulation demonstrated a size of 123.40 ± 2.00 nm and a PDI of 0.2099 ± 1.00, suggesting a monodisperse system with a single peak observed in DLS analysis (Figure 2A). The International Standard Organization (ISO) guidelines suggest that formulations with a PDI exceeding 0.7 are prone to aggregation [43]. For lipid-based drug delivery, a PDI of 0.3 or less is considered appropriate [34]. Furthermore, the zeta potential values of investigated CBD-SLNs have been found to range between −33.13 and −28.23 mV depending on the specific composition. Under optimal conditions, the SLN formulation exhibited a zeta potential of −31.25 ± 0.21 mV (Figure 2B). Maintaining a surface charge of at least ±30 mV is crucial for stable colloidal dispersions through electrostatic repulsion between particles [44]. These CBD-SLNs were spherical, as depicted by transmission electron microscopy (TEM) (Figure 2C,D). The EE and DL of nanoparticles represent their capacity to efficiently incorporate and retain a substantial amount of the drug within their structure. In the case of CBD-SLNs, it exhibited a high EE of 95.16 ± 0.14 and DL of 2.36 ± 0.05.

The X-ray diffractometer (XRD) patterns of free CBD, lipid, blank SLNs, and CBD-SLNs are depicted in Figure 2E. The comparison between CBD and CBD-SLNs diffraction indicates a notable distinction. The analysis of pure CBD displayed a sequence of sharp diffraction peaks between 2θ values of 5° and 30°, consistent with various research findings that show its crystalline properties [45,46]. Importantly, these characteristic peaks were not observed in the XRD pattern of the CBD-SLNs. This suggests that the CBD was effectively dissolved within the lipid matrix of the SLNs and stabilized in an amorphous state. Figure 2F displays the FTIR spectra of pure CBD, CBD-SLNs, blank SLNs, and GMS, aiding in identifying the stability and interactions of CBD-SLNs. Fourier-transform infrared spectroscopy (FTIR) is a well-established method employed in pharmaceutical formulations to analyze interactions between drugs and excipients or detect chemical incompatibilities. When a drug and an excipient interact or incompatibility arises, discernible changes manifest in the infrared spectra. These alterations encompass shifts in peak positions, variations in intensity, or the emergence/disappearance of specific peaks. The comprehensive analysis of these spectral changes is instrumental in identifying potential issues that may contribute to the drug’s instability or diminished efficacy within the formulation [47,48]. The FT-IR spectrum of CBD exhibits distinctive molecular vibrations within specific regions: 3405–3518 cm^−1^ for O–H (aromatic) stretching, 3000 cm^−1^ for C–H stretching (phenyl), 2923 cm^−1^ for methyl/methylene groups, 1581 cm^−1^ for C=C stretching (phenyl ring), and 1214 cm^−1^ for C–O stretching vibrations [49]. Notably, some bands in CBD-SLNs and CBD overlap with lipid/GMS bands, such as the 2914 cm^−1^ peak representing –CH2 and –CH3 groups’ stretching vibration and the 1195–1219 cm^−1^ bending vibration of C–O. [50,51]. Pure CBD’s distinct peaks at 3000–3518 cm^−1^ are absent in GMS and blank SLNs, indicating their absence. Moreover, the peaks attributed to CBD in the CBD-SLNs exhibit slight shifts: from 3518 to 3517 cm^−1^, 3405 to 3402 cm^−1^, and 2923 to 2915 cm^−1^. It is important to note that the absence of new peaks and the lack of significant changes in the characteristic CBD peaks further demonstrate the excellent compatibility of CBD with the lipidic matrix and the successful incorporation of the bioactive compounds into the NP structures, consistent with findings reported by Verdanega et al. [52] and Matarazzo et al. [53] in their CBD-loaded lipid NPs.

### 2.4. In Vitro Release Study

**Figure 3:**
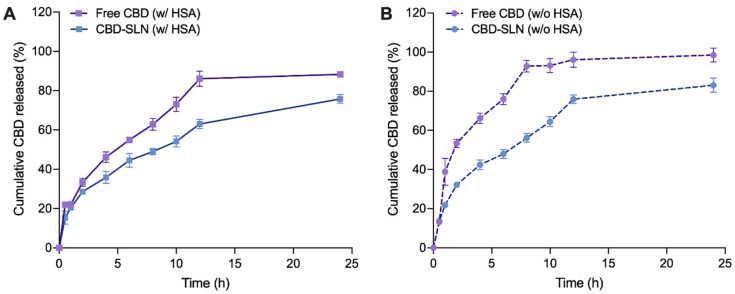
Cumulative drug release of CBD over 24 h at physiological pH (7.4). (**A**) With and (**B**) without the presence of human serum albumin (HSA). Data are presented as the mean ± SD (n = 3).

In assessing the release profile of CBD from the SLNs, it was crucial to consider the molecular weight of CBD relative to the MWCO of the dialysis membrane used in our study. CBD, with a molecular weight of approximately 314.47 g/mol, required a dialysis membrane capable of effectively distinguishing between the encapsulated compound and the free drug released from the SLNs. Therefore, we employed a dialysis membrane with an MWCO of 3.5 kDa (equivalent to 3500 g/mol), allowing unencapsulated CBD to freely pass through while retaining the larger SLN particles within the dialysis bag. This methodological choice enabled a precise evaluation of CBD release dynamics from the NPs, aligning with standard protocols for NPs drug release studies [54,55]. The CBD release study from the SLNs was conducted to evaluate release behavior and kinetics in the presence of proteins. In the presence of human serum albumin (HSA), free CBD leads to a notably higher cumulative release of 86.08% after 12 h and 96.00 after 24 h (Figure 3A). In contrast, CBD-SLNs exhibit a distinct release pattern, characterized by a sustained release profile. The cumulative CBD release percentages for CBD-SLNs were markedly lower, at 63.01% and 75.83% after 12 and 24 h, respectively. Moreover, it can be noted that the free CBD exhibited higher release rates than CBD-SLNs over 24 h, considering that CBD has low water solubility. This difference can be attributed to the specific composition of the release medium, which contained a surfactant and a 70:30 mixture of PBS and ethanol. These components significantly enhanced the solubility of free CBD, promoting its rapid dissolution and diffusion in the surrounding medium. Conversely, CBD-SLNs are expected to provide controlled and sustained release characteristics. CBD encapsulated within the SLNs must diffuse out of the lipid matrix, leading to a slower release profile than freely dissolved CBD. The lipid matrix of the SLNs acts as a barrier that governs the rate of CBD release into the release medium, resulting in a more controlled and prolonged release pattern. To further substantiate these findings, De Gaetano et al. [56] and Hassan et al. [57] have reported similar observations regarding the controlled release behavior of non-polar compounds from SLNs. These studies reinforce our understanding of how formulation strategies can influence the release characteristics of compounds like CBD, underscoring the importance of customized delivery systems in enhancing CBD’s therapeutic effectiveness. In this study, HSA significantly influences CBD release profiles. Without HSA, CBD suspension reached 96.08% at 12 h and 98.48% at 24 h (Figure 3B). In contrast, CBD-SLNs had lower cumulative release: 75.95% at 12 h and 83.04% at 24 h. This suggests that, in the absence of HSA, higher levels of CBD can be released from both samples under investigation.

The cumulative release percentages vary due to intricate interactions among CBD, the lipid carrier, and HSA, impacting the drug release mechanism [58]. HSA, a prominent plasma protein, is known for its capacity to bind and transport various substances, including drugs [59]. CBD binds to HSA, forming the CBD-HSA complex, which affects the solubility and diffusion of CBD [60,61]. Typically, HSA binding restricts drug diffusion, diminishing the release of CBD from delivery systems such as suspensions or SLNs [62]. Furthermore, the presence of HSA on the surface of CBD-SLNs can enhance protein adsorption, thereby forming a barrier that impacts the release of CBD from these nanoparticles. Additionally, the adsorption of HSA onto the dialysis membrane can alter the drug’s permeability and create resistance to diffusion. To avoid the interaction of the drug with HSA inside the dialysis bag, which might block the penetration of the released CBD through the membrane due to CBD’s high affinity for HSA, we purposely did not include HSA inside the dialysis bag in this study. This approach was deliberately chosen, considering that HSA, with an MW of 66.5 kDa, binds to CBD, forming the CBD-HSA complex, which cannot traverse the dialysis membrane with an MWCO of 3.5 kDa. Studies have shown that buffers containing additives such as surfactants, salts, or chaotropic agents can significantly reduce protein adsorption on dialysis membranes [63,64]. Notably, we integrated surfactant poloxamers into the release media in our experiment, thereby minimizing the potential adsorption of HSA onto the dialysis membrane.

To elucidate CBD release patterns from the SLNs, data were fitted into different empirical models, including zero-order, first-order, Higuchi, Korsmeyer–Peppas, and Hixson–Crowell models, using the DDsolver add-in program in Microsoft Excel 2010 (Tables S6 and S7) [65]. The optimal model was chosen based on a high R^2^ value close to 1, a high model selection criterion (MSC), and a low Akaike information criterion (AIC) [66]. The Korsmeyer–Peppas model best fit most samples, except for free CBD without HSA, where the first-order mechanism was optimal. Release mechanisms were determined by diffusional index (n) values: n < 0.45 indicates quasi-Fickian diffusion, n = 0.45 signifies Fickian release, 0.45 < n < 0.89 suggests non-Fickian/anomalous release, n = 0.89 represents case II/zero-order release, and n > 0.89 indicates super case II release (Table 4) [67]. All samples fitting the Korsmeyer–Peppas model exhibited quasi-Fickian diffusion, consistent with prior studies on nanoparticle formulations, particularly lipid-based nanoparticles [68].

Our in vitro release study acknowledges the differences between laboratory conditions and the physiological environment, posing challenges to directly applying our findings in a clinical context [69]. While our results demonstrate a promising sustained release profile of CBD from SLNs, it is important to note that our study focused on oral administration. The sustained release observed in our SLN formulation offers significant therapeutic potential for systemic conditions, such as chronic inflammatory diseases, where continuous drug delivery can enhance treatment efficacy by maintaining consistent drug levels over time. This sustained release mechanism is particularly advantageous for conditions requiring prolonged therapeutic intervention, providing a more controlled and effective delivery of CBD through oral administration. Factors such as metabolic processes, elimination pathways, and interactions with biological matrices can profoundly influence the pharmacokinetics and efficacy of the drug [70]. Therefore, to validate our initial in vitro findings and evaluate the clinical relevance of CBD-SLNs, comprehensive in vivo studies are essential. These investigations should involve examining the pharmacokinetics, distribution, and therapeutic efficacy of CBD-SLNs in vivo. Bridging the gap between in vitro and in vivo data is crucial for the clinical translation of our research, emphasizing the need for in vivo experiments to confirm the potential of CBD-SLNs as a sustained-release drug delivery system for conditions that benefit from ongoing CBD therapy.

### 2.5. Stability of CBD-SLNs

**Figure 4:**
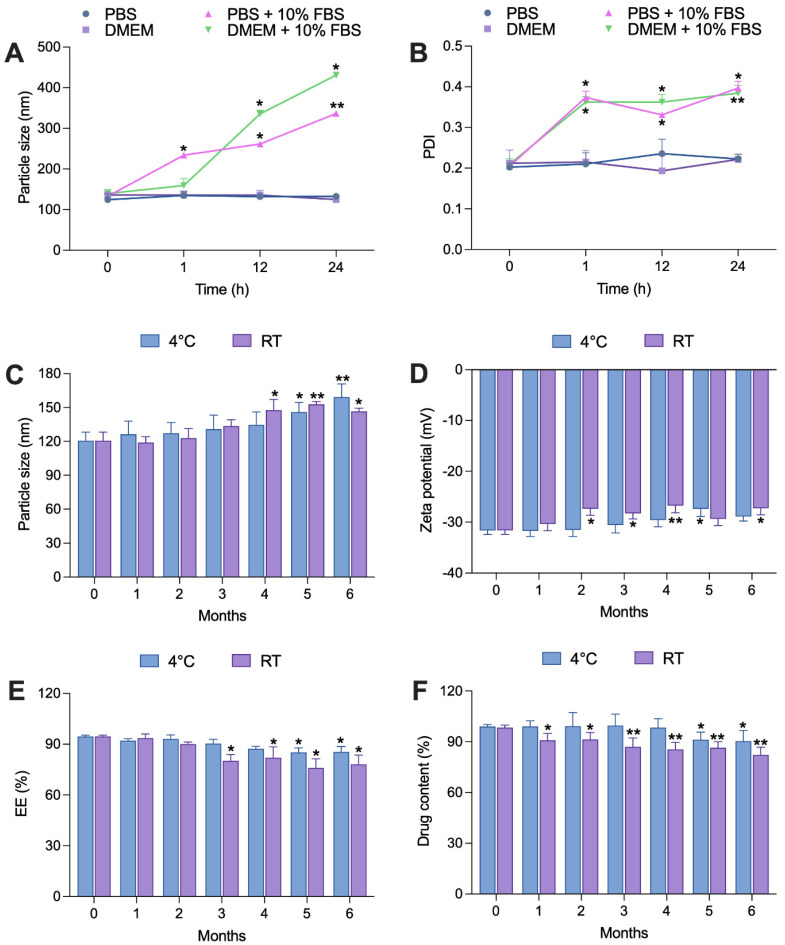
Stability studies of CBD-SLNs. (**A**,**B**) Colloidal stability in biologically relevant media. (**C**–**F**) Storage stability at 4 °C and room temperature (RT) for 6 months. Data are presented as the mean ± SD (n = 3). * *p* < 0.05 and ** *p* < 0.001 compared to formulation characteristics on day 0.

Stability tests were focused on the aqueous dispersion of SLNs, chosen for its relevance to intended oral applications. The colloidal stability of nanoparticles in biologically relevant media was assessed by examining the size (Figure 4A) and PDI (Figure 4B) before conducting cell assays. The culture medium used in these experiments contained various biomolecules, including proteins like serum albumin and globulins, along with amino acids and ionic salts, all of which can significantly affect nanoparticle behavior [71,72]. CBD-SLN samples showed consistent size and PDI in phosphate buffer solution (PBS) and Dulbecco’s modified eagle medium (DMEM), but changes occurred with 10% fetal bovine serum (FBS), aligning with the release study results. Serum affects CBD-SLN release and characteristics via protein interactions. To mitigate this, serum-free media were employed in future experiments, improving nanoparticle control in biological applications.

The long-term physical stability of CBD-SLNs was also assessed over 6 months to check for particle aggregation or drug leakage during storage. CBD-SLNs stored at 4 °C for up to 4 months showed no significant changes in size (Figure 4C), zeta potential (Figure 4D), EE (Figure 4E), or drug content (Figure 4F). In the 5th month, size increased, indicating aggregation, with a higher PDI. EE and drug content dropped by 9% and 7%, possibly due to drug diffusion from the lipid matrix. CBD-SLNs stored at room temperature (RT) had significant changes earlier. By the 4th month, size increased, surface charge decreased, and there was a 14% EE reduction. Temperature fluctuations and humidity during storage likely caused physical instability, potentially leading to drug expulsion and reduced EE. Additionally, chemical degradation of the drug or lipid components over time could impact overall drug content and EE.

Overall, storing CBD-SLNs at 4 °C is favorable for stability, underlining the importance of proper storage conditions to maintain formulation characteristics [73]. These insights are valuable for CBD-SLN development and storage in pharmaceutical or food applications.

### 2.6. In Vitro Cellular Assay

Inflammation is a major contributor to the pathogenesis of several chronic inflammatory conditions, including but not limited to arthritis, atherosclerosis, and osteoarthritis (OA). This pathological process activates innate immunity, leading to the generation of cytokines such as IL-1β, IL-6, IL-8, and TNF-α [74]. We utilized an in vitro cell model of OA as a representative model to screen for the potential of CBD-SLNs for the treatment of chronic inflammatory conditions. Indomethacin was used as a reliable positive control in the in vitro studies to evaluate anti-inflammatory properties and ensure the reliability of the results (see Supplementary Figures S5 and S6). In this study, we employed serum-free media to prevent potential interactions between serum proteins and the treatments. This precaution ensures that the observed effects are more likely attributed to the compounds tested rather than potential interactions with serum components [75]. The removal of serum also eliminates potential interference with the compound’s activity. This approach aids in accurately assessing the direct impact of the compound on the cells, facilitating a clearer interpretation of the assay results [76,77].

#### 2.6.1. Effects of CBD-SLNs on Cell Viability of Proinflammatory Cytokine-Stimulated Chondrocytes and Macrophages

**Figure 5:**
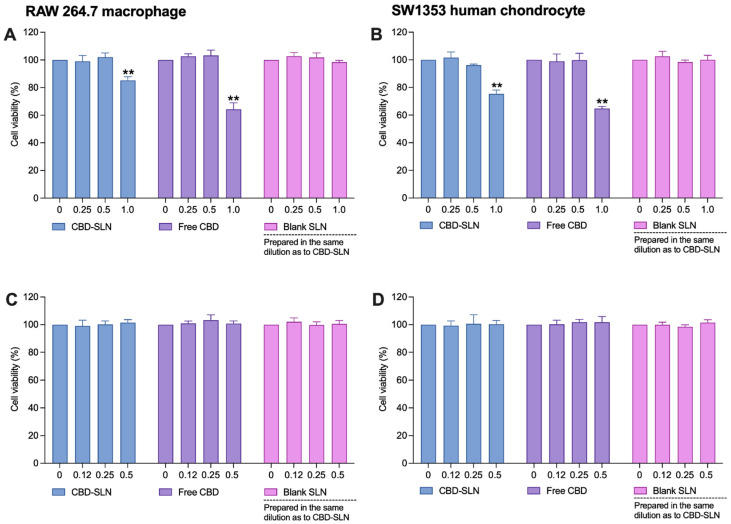
Cytotoxic effects of the CBD-SLNs, free CBD, and blank SLNs on (**A**,**B**) RAW 264.7 murine macrophages and SW 1353 human chondrocytes and (**C**,**D**) LPS-stimulated RAW 264.7 murine macrophages and IL-1β-stimulated SW 1353 human chondrocytes. Data are presented as the mean ± SD (n = 3). ** indicates a significant difference (*p* < 0.01) compared to the untreated cells (0 µg/mL).

The cytotoxicity of CBD and CBD-SLNs on SW 1353 and RAW 264.7 cells was assessed at concentrations of 0.25–1 μg/mL. Both showed cytotoxicity at 1 μg/mL, while blank SLNs at equivalent dilutions to CBD-SLNs had no toxicity (Figure 5A,B). To further validate the safety of concentrations < 0.5 μg/mL, we conducted an additional cell viability experiment on proinflammatory cytokine-stimulated cells. The SW 1353 and RAW 264.7 cells were stimulated with inflammatory inducers, IL-1β and lipopolysaccharides (LPS), respectively, for 24 h. As shown in Figure 5C,D, none of the treatments exhibited significant toxicity to the stimulated cells. Consequently, we can confidently proceed with these concentrations for subsequent experiments to further evaluate their anti-inflammatory effects.

#### 2.6.2. Effects of CBD-SLNs on the Inhibition of Cellular Free Radical Generation and Secretion of Inflammatory Components in LPS-Stimulated RAW 264.7 Macrophages

In this study, we investigated the impact of CBD-SLNs on inhibiting cellular reactive oxygen species (ROS), nitric oxide (NO), TNF-α, and IL-6 levels in proinflammatory cytokine-stimulated cell lines. The reactive nitrogen species (RNS), including NO, serve as a key proinflammatory mediator, IL-6 acts as a major proinflammatory cytokine, and TNF-α is the earliest endogenous mediator of inflammatory reactions. ROS plays a role in the degradation of tissues associated with inflammation in OA and is typically described as partially reduced byproducts of oxygen possessing potent oxidative properties [78].

**Figure 6:**
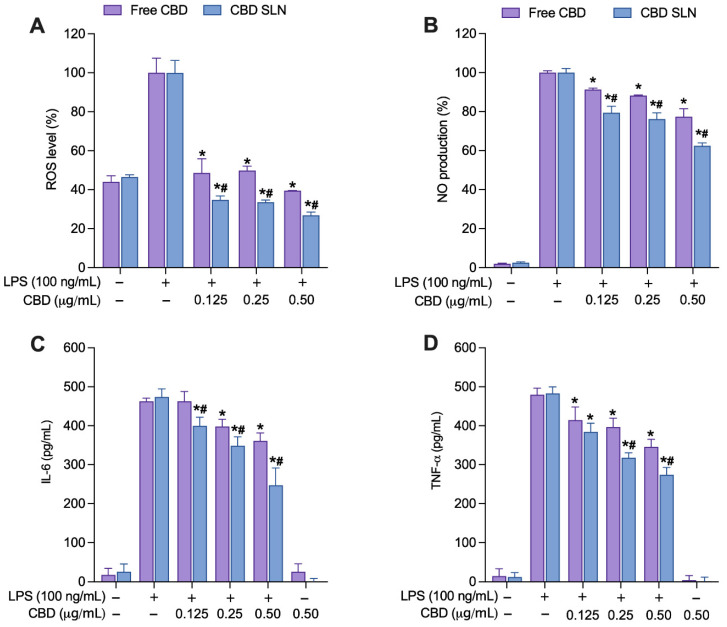
The effects of CBD-SLNs and free CBD on (**A**) cellular ROS and (**B**) NO production and the suppression of proinflammatory cytokines (**C**) IL-6 and (**D**) TNF-α in LPS-stimulated RAW 264.7 macrophages. Data are presented as the mean ± SD (n = 3). * *p* < 0.05 and ^#^ *p* < 0.05 compared to LPS-stimulated cells without treatment and to free CBD, respectively.

Non-cytotoxic concentrations (0.125–0.50 μg/mL) of CBD-SLNs and free CBD were co-incubated with 100 ng/mL of LPS for 24 h in RAW264.7 cells. CBD-SLNs exhibited a significant reduction in ROS compared to both untreated LPS-stimulated cells and free CBD (Figure 6A). At the highest concentration tested (0.50 μg/mL), free CBD and CBD-SLNs displayed ROS levels of 40% and 27%, respectively. This equates to a substantial ROS inhibition of 60% (free CBD) and 73% (CBD-SLNs) compared to the untreated, stimulated-cell control. The nitrite assay revealed an apparent concentration-dependent reduction in NO production, with the highest concentration of 0.50 μg/mL exhibiting the most significant inhibition for both treatments (Figure 6B). Additionally, statistical analysis revealed a significant suppression in NO production in the CBD-SLN treatment group compared to free CBD (*p* < 0.05). At a 0.50 μg/mL concentration, free CBD and CBD-SLNs demonstrated NO production levels of 77% and 62%, respectively. These data highlight that CBD-SLNs have a 1.2-fold greater potency than free CBD.

In enzyme-linked immunosorbent assay (ELISA) tests quantifying TNF-α and IL-6 levels, CBD-SLNs consistently displayed stronger, concentration-dependent cytokine inhibition compared to free CBD (Figure 6C,D). CBD-SLNs significantly reduced IL-6 even at the lowest concentration (0.125 µg/mL), while free CBD exhibited IL-6 reduction at 0.25 µg/mL (Figure 6C). At 0.50 µg/mL, CBD-SLNs were 1.5-fold more effective than free CBD. For TNF-α inhibition, both CBD-SLNs and free CBD had substantial effects at all concentrations (0.125–0.5 µg/mL) (Figure 6D), with CBD-SLNs consistently outperforming free CBD. At the highest concentration (0.50 µg/mL), CBD-SLNs exhibited 1.3-fold greater potency, highlighting its promising therapeutic potential for inflammation management.

#### 2.6.3. Effects of CBD-SLNs on the Suppression of Cellular Free Radical Generation and Proinflammatory Cytokine Levels in IL-1β-Stimulated SW 1353 Chondrocytes

**Figure 7:**
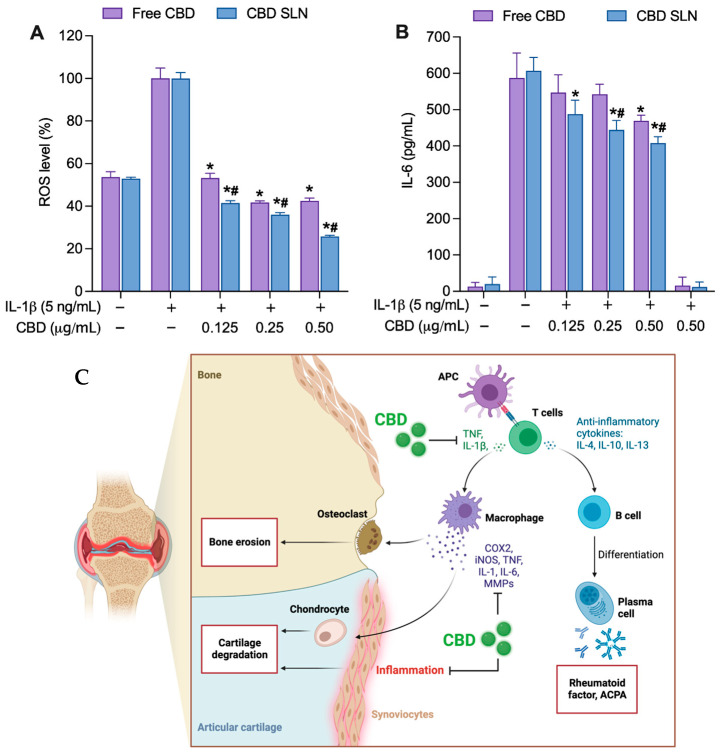
The effects of CBD-SLNs and free CBD on (**A**) cellular ROS and (**B**) IL-6 in IL-1β-stimulated SW 1353 cell lines. Data are presented as the mean ± SD (n = 3). * *p* < 0.05 and ^#^ *p* < 0.05 compared to IL-1β -stimulated cells without treatment and to free CBD, respectively. (**C**) A general illustration of the signaling pathways involved in the anti-inflammatory effects of CBD in OA progression. Abbreviations: APC, antigen-presenting cells; IL, interleukin; TNF, tumor necrosis factor; COX, cyclooxygenase; iNOS, inducible nitric oxide synthase; MMP, matrix metalloproteinase.

Human chondrocyte SW 1353 is a widely used in vitro model for OA. Typically, IL-1β or TNF-α induces an inflammatory and catabolic response, increasing MMPs and proinflammatory cytokine production [79]. Based on previous studies, IL-1β can significantly stimulate ROS and IL-6 in SW 1353 cells. The augmented ROS generation in chondrocytes activates the release of inflammatory responses, primarily IL-6 [80]. The IL-6 signaling was studied as one of the major cytokines that mediate cartilage degradation and pain in OA through the Janus kinases (JAKs) pathway [80]. Non-cytotoxic concentrations (0.125–0.50 μg/mL) of CBD-SLNs and free CBD were co-incubated with 5 ng/mL of IL-1β for 24 h in SW 1353 cells. Both formulations significantly reduced cellular ROS production across all concentrations (Figure 7A), with CBD-SLNs showing greater inhibitory activity than free CBD. At the highest concentration (0.50 µg/mL), free CBD and CBD-SLNs reduced ROS levels to 43% and 26%, respectively, corresponding to 57% (free CBD) and 74% (CBD-SLNs) ROS inhibition relative to the untreated, stimulated-cell control. CBD-SLNs displayed concentration-dependent IL-6 reduction in IL-1β-stimulated cells, similar to its behavior in LPS-stimulated macrophages. Free CBD showed noticeable effects only at the highest 0.50 μg/mL concentration (Figure 7B). At this level, CBD-SLNs demonstrated a significant 1.1-fold increase in IL-6 inhibition compared to free CBD. These results emphasize CBD’s enhanced bioactivity when encapsulated in SLNs and its potential for targeted therapeutic applications, especially in managing IL-6-mediated responses in SW 1353 cells.

In summary, CBD-SLNs outperform free CBD in suppressing ROS and cytokine production in LPS-stimulated macrophages and IL-1β-stimulated SW 1353 chondrocytes. Even without LPS/IL-1β treatment, cells showed basal ROS production, lower than treated cells. CBD-SLNs and free CBD concentration-dependently inhibited ROS, with CBD-SLNs showing significantly more potent inhibition. These results offer insights into CBD’s modulation of ROS production, particularly in LPS and IL-1β-induced oxidative stress in macrophages and chondrocytes. Excessive ROS arises from oxidative stress due to an imbalanced antioxidant defense system, contributing to inflammatory arthritis and potentially leading to OA [81]. Inflammation induces hypoxia in synovial cells, causing mitochondrial damage and increased ROS levels, worsening synovitis. Oxidative stress also accelerates telomere shortening and chondrocyte aging, leading to OA onset by disrupting mitochondrial redox regulation and triggering ROS production, exacerbating OA symptoms [82].

Figure 7C provides a general illustration of the signaling pathways involved in CBD anti-inflammatory effects in OA progression [2,83]. This research provides evidence that the nanoencapsulation of CBD as CBD-SLN formulation markedly improves CBD efficacy in modulating the immune response, leading to a significant reduction in the production of proinflammatory cytokines and ROS. Exploring the molecular mechanisms, our study reveals that CBD encapsulated in solid lipid nanoparticles (SLNs) effectively modulates key biochemical pathways associated with inflammation and oxidative stress. Research, including studies by Atalay et al. (2019) [4] and Jitca et al. (2023) [84], highlights CBD’s impact on various signaling pathways—particularly its role in reducing proinflammatory cytokines and enhancing the body’s antioxidant defenses. It interacts with various molecular targets, including cannabinoid receptors and other endocannabinoid system components, and has been shown to engage different targets, such as GPCRs and ion channels [85]. The endocannabinoid system, which CBD interacts with, plays a crucial role in regulating biological processes and has been targeted for the development of therapeutics for cannabis use disorders. Such mechanisms are crucial for managing chronic inflammatory conditions, where these pathways are significantly involved. Compared to traditional CBD formulations, our CBD-SLNs offer controlled release and improved bioavailability, significantly enhancing therapeutic efficacy, as demonstrated by Eydelman et al. (2023) [86] and Zielinska et al. (2023) [15]. Moreover, the benefits of our NP delivery system extend further, as evidenced by comparative analyses with other nanoformulations in recent scientific literature, which suggest superior stabilization and bioactivity—findings that align with those of Ganesan et al. (2018) [87], Fonseca-Santos et al. (2020) [88], and Viegas et al. (2023) [89]. These discussions enrich our understanding of the therapeutic potential of CBD-SLNs, positioning our research within the wider scientific context and highlighting the innovative nature of our approach. Compared with traditional pharmaceutical forms, CBD-SLNs emphasize both the benefits and challenges of this novel formulation. CBD-SLNs offer enhanced bioavailability and controlled drug release, which is crucial for chronic conditions requiring consistent therapeutic levels [15,86,88,89]. Additionally, the nanoparticle formulation reduces cytotoxicity associated with higher doses of free CBD. However, manufacturing CBD-SLNs involves complex techniques like hot homogenization and ultrasonication, which could elevate production costs and complicate scaling up for commercialization. Stability over long periods and under varying conditions also poses a significant challenge alongside the stringent regulatory scrutiny that nanoparticle-based systems typically face [18,20]. These factors must be carefully considered as we advance the development and potential commercial application of CBD-SLNs

In this study, we have acknowledged the inherent limitations of in vitro investigations. Consequently, further in vivo research is imperative to validate our conclusions and to elucidate the clinical applicability of CBD-SLNs. Such research bridges the gap between promising laboratory findings and actual clinical benefits. It will facilitate a comprehensive understanding of the therapeutic potential, pharmacokinetics, and safety profile of CBD-SLNs, laying the groundwork for their potential use in treating chronic inflammatory conditions.

## 3. Materials and Methods

### 3.1. Chemicals and Materials

Cannabidiol (CBD isolate, 98% purity) was obtained from Inspector and Engineering Co., Ltd. (Bangkok, Thailand). Glyceryl monostearate (GMS) was obtained from S. Tong Chemicals Co., Ltd. (Nonthaburi, Thailand). Polysorbate 80 was acquired from Thermo Fisher ACROS Organics™ (Geel, Belgium). Ethanol, methanol, sodium hydroxide, sodium acetate, potassium dihydrogen phosphate, dipotassium hydrogen phosphate, sodium chloride, and others were purchased from Carlo Erba (Val de Reuil, France). Deionized water and ultra-pure water were prepared in-house and used freshly. All solvents were of at least analytical grade and used without further purification.

### 3.2. Extraction, Isolation, and Purification of CBD

The inflorescence of *Cannabis sativa* L. subsp. Sativa was obtained from Chiang Rai Province, Thailand, and authenticated by one of the authors (W.T.). The hemp inflorescence was dried at 50 °C and pulverized into powder before extraction. CBD extraction was performed using a customized in-house method to maximize yield and purity. The CBD powder was derived from hemp plants through a cold ethanol extraction process. Initially, 1 kg of mature hemp plants was harvested and dried to minimize moisture content. Subsequently, the dried hemp material was finely ground to augment the surface area, facilitating enhanced solvent penetration. The finely ground plant material was then introduced into an in-house extractor, and cold ethanol (maintained between −35 and −40 °C) was added to the extraction machine in a 1:1 ratio (weight of dried plant to the volume of ethanol). Ethanol at temperatures ranging from −30 °C to −40 °C was utilized for extraction. Specifically, ethanol at a significantly lower temperature range was selected due to its efficacy in solubilizing CBD while minimizing the extraction of unwanted nonpolar compounds, such as chlorophylls and waxes, which exhibit less solubility at lower temperatures [90]. Notably, waxes can impede CBD crystallization during recrystallization, potentially leading to the observed low final yield after purification. The mixture was stirred at 50 rpm for 30 min, allowing the cold ethanol to dissolve cannabinoids and other desired components. The extraction process was conducted while maintaining the temperature between −35 and −40 °C. The liquid extract was concentrated using a rotary evaporator to remove the ethanol. The concentrated oil and ethanol underwent freezing to eliminate unwanted waxes and lipids, which solidify during freezing. The resultant mixture was filtered to eliminate solidified impurities. The winterized oil underwent further concentration to yield a more refined oil. This refined oil was heated to convert any remaining CBDA to CBD. The decarboxylated CBD oil was subjected to fractional distillation for purification, resulting in more concentrated CBD distillates. CBD crude powder was isolated from CBD distillates through crystallization in pentane. The crude powder underwent recrystallization to achieve high-purity CBD, resulting in a purity greater than 99.5% (40.5 g). The yield was about 4% from dried hemp, IR (KBr): υ_max_ 3405–3518 (O–H (aromatic)), 3000 (C–H (alkene)), 2923 (C–H (alkane)), 1581 (C=C (phenyl)), 1214 (C–O) cm^−1^ (Supplementary Figure S1); ^1^H NMR (CDCl_3_, 500 MHz): δ 0.86 (3H, t, J = 6.8 Hz), 1.29 (4H, m), 1.54 (2H, q, J = 7.6 Hz), 1.64 (3H, s), 1.79 (3H, s), 1.83 (2H, m), 2.09 (1H, m), 2.24 (1H, m), 2.40 (1H, m), 2.43 (2H, t, J = 7.4 Hz), 3.84 (1H, dm, J = 11.8 Hz), 4.54 (cis, 1H, m), 4.64 (trans, 1H, m), 4.82 (1H, s(br)), 5.55 (1H, s), 5.97 (1H, s(br)), 6.16 (1H, s(br)), 6.25 (1H, s(br)) (Figure S2); ^13^C NMR (CDCl_3_, 125 MHz): δ 13.99, 20.39, 22.50, 23.64, 28.35, 30.35, 30.60, 31.46, 35.44, 37.13, 46.14, 107.96, 109.68, 110.82, 113.73, 124.105, 139.99, 142.99, 149.25, 153.89, 155.99 (Figure S3); and HRMS (*m*/*z*): 337.21309 (C_21_H_30_NaO_2_) [M + Na^+^] (Figure S4).

### 3.3. Preparation of CBD-Loaded SLNs

The formulation of our CBD-SLNs was meticulously developed through initial experiments aimed at identifying the optimal lipid matrix. This process was underpinned by an extensive review of the literature focusing on lipid-based NPs for drug delivery, emphasizing their physicochemical characteristics, biocompatibility, and performance in encapsulating hydrophobic compounds like CBD [91,92]. GMS is widely utilized in lipid nanoparticle development, notably in SLNs, owing to its favorable properties and safety profile. Numerous studies have highlighted GMS’s efficacy in enhancing drug solubility and achieving controlled drug release of hydrophobic compounds [93,94,95]. The choice of GMS as the lipid component for our SLN formulation was determined by assessing the solubility of CBD. GMS was considered a suitable candidate due to the absence of drug crystals; the visual inspection of CBD solubility in melted lipids confirmed GMS’s ability to dissolve CBD completely [96,97,98]. The CBD-SLNs were prepared using low-energy hot homogenization and ultrasonication methods described by Kaisit et al., with some modifications [97]. In brief, food-grade GMS lipid, considered GRAS (generally recognized as safe), was melted with 3 mL ethanol at 80 °C. Simultaneously, polysorbate 80 was mixed with 80 mL of water at the same temperature and mixed at 1000 rpm for 10 min. Methanolic CBD (1 mL) was added to the melted lipid and stirred for 3 min. The lipid phase was then added to the aqueous phase, resulting in emulsion formation, and was continuously mixed for 30 min. Hot water was added to maintain the volume at 100 mL due to evaporation during mixing. A bath sonicator (Powersonic™ P230D, Crest Ultrasonics, NJ, USA) was used for 30 min to reduce emulsion size further. The colloidal dispersion was cooled at room temperature (RT) and left overnight to ensure complete lipid crystallization.

### 3.4. Experimental Design for the Optimization of CBD-SLNs

BBD is a valuable statistical tool with a three-factor, three-level design. In this study, critical parameters for CBD-SLN formulation were lipid/GMS (A), surfactant/polysorbate 80 (B), and CBD amount (C), chosen based on initial experiments. A three-level BBD with three replicated center points for each factor was used to optimize the formulation and assess its impact on particle size, PDI, % EE, and % DL (Table 1). Design-Expert^®^ version 23.1 (Stat-Ease^®^ Inc., Minneapolis, MN, USA) generated 15 formulation conditions (Table 2). ANOVA validated the statistical significance of polynomial equations. Three-dimensional response surface and contour plots from the same software identified the optimal formulation. Predicted values were compared to experimental results to assess optimization accuracy. The experimental design process involved utilizing a BBD to generate a comprehensive set of experimental conditions encompassing varying levels of GMS, polysorbate 80, and methanolic CBD. This allowed for systematically exploring formulation combinations on specific SLN characteristics such as size, PDI, EE, and DL. Subsequently, the responses obtained from these experiments, including size, PDI, EE, and DL, were subjected to regression analysis and model fitting followed by graphical interpretation to discern individual factors’ impact and interactions on the desired SLN properties. By analyzing response surface plots and contour maps derived from the BBD, optimal formulation conditions were identified that concurrently optimized the targeted SLN characteristics essential for effective CBD delivery. Finally, these optimized conditions were rigorously validated through experimental verification to ensure the reproducibility and robustness of the formulated CBD-SLNs, consolidating the reliability of the study outcomes.

### 3.5. Physicochemical Characterization of CBD-Loaded SLNs

The aqueous dispersion of CBD-SLN was diluted directly in normal saline for size and PDI measurements using dynamic light scattering (DLS, Nano-ZS, Malvern PANalytical Ltd., Malvern, UK). The diluted SLN dispersion was gently stirred to ensure uniform mixing before the measurements were conducted. No ultrasonication was used during the dilution process to minimize potential disruption of the SLN structure. At the same time, the zeta potential was determined by analyzing its electrophoretic mobility using the same equipment. The morphology of the CBD-SLNs was observed through TEM (JEM-1400, JEOL Ltd., Tokyo, Japan).

The DL (Equation (6)) and EE (Equation (7)) of the CBD-SLNs were determined by measuring the dry weight of the separated NPs and the absorbance of the supernatant, respectively. The CBD-SLNs were ultracentrifuged at 4 °C at 105,000× *g* for 1 h. The absorbance was then measured at 210 nm (Agilent Cary 60, Agilent Technology, Santa Clara, CA, USA), and the amount of free CBD in the supernatant was calculated against the standard curve. The DL and EE of the CBD-SLNs were calculated using the following equations:

**DL (%)** = [(Wt − Ws)/Wnp)] × 100 (6)

**EE (%)** = [(Wt − Ws)/Wt)] × 100 (7)

where Wt is the initial amount of CBD added to the formulation, Ws is the amount of free CBD in the supernatant, and Wnp is the weight of the CBD-SLNs after lyophilization. The lyophilization process was performed after removing the supernatant and collecting the SLN at the bottom. The NPs were frozen for 5 min at −80 °C to solidify the water content rapidly and then subjected to freeze-drying under vacuum using the FreeZone Freeze Dry System (Labconco Corp., Kansas City, MO, USA) at −55 °C for 24 h and weighed accordingly.

IR spectra and XRD patterns of CBD-SLNs, blank SLNs, and their components were analyzed to assess CBD encapsulation and crystallinity post-preparation. FTIR spectra ranged from 4000 to 400 cm^−1^ (Spectrum one™, PerkinElmer Inc., Waltham, MA, USA). XRD patterns were obtained with a wide-angle XRD (PANalytical X’Pert Pro model, PANalytical, Kassel-Waldau, Germany) scanning at 0.05°/min from 3 to 70° at room temperature.

### 3.6. In Vitro Release Study

The release of CBD from an SLN formulation was investigated in vitro using the dialysis bag method designed for precision and reproducibility. In the release test, CBD-loaded SLNs were enclosed in a dialysis bag (MWCO: 3.5 kDa) to isolate them from the release medium. The release medium was a blend of phosphate-buffered saline (PBS), ethanol (70:30), and 3% Poloxamer (pH 7.4), selected to ensure CBD solubility while mimicking physiological conditions to some extent. A total of 5 mL of the dispersion (CBD suspension or aqueous dispersion of CBD-SLNs) was sealed in a dialysis bag and placed in 200 mL of pH 7.4 release medium at 37 ± 0.5 °C with continuous stirring at 50 rpm to maintain sink conditions throughout the experiment. This setup facilitated the diffusion of released CBD without saturating the medium. Thus, solubility studies were conducted prior to the release tests to confirm that CBD’s solubility surpassed its concentration in the release medium throughout the experiment, ensuring sink conditions. With a solubility exceeding 30 μg/mL, this step was crucial for accurately simulating drug release from the SLNs, preventing saturation, and facilitating the diffusion process effectively. To further study the interaction of SLN with proteins, Human Serum Albumin (HSA) was introduced to the external medium, considering its stability in 30% ethanol [99,100]. Additionally, 1 mL aliquots were collected at set intervals and replaced with fresh release medium. CBD concentration was measured using spectrophotometry to calculate % cumulative CBD released using the formula below (Equation (8)):

(8) $$\mathrm{CR}\;\left(\%\right)=\frac{V_{e}\sum_{i=1}^{n-1}C_{n-1}+V_{o\;}C_{n}}{m}\times 100$$

where *CR* (%) is cumulative CBD released, *V_e_* is collection volume (mL), *V_o_* is the volume of the medium (mL), Cn is the concentration of the time point (mg/mL), and m is total CBD (mg) in the dialysis bag. Finally, the release profiles of the samples were subjected to mathematical modeling using several well-established equations, including the zero-order, first-order, Korsmeyer–Peppas, Hixon–Crowell, and Higuchi equations [101].

### 3.7. Storage and Colloidal Studies

The storage stability of the CBD-SLNs was investigated over six months by conducting regular evaluations of the nanoparticle’s size, total drug content, zeta potential, and EE. The formulations were stored in light-resistant glass vials at two different temperatures: 4 °C and room temperature [102]. In our investigation into the stability of SLN formulations, we opted to store the formulations as aqueous dispersions within light-resistant glass vials. This decision was predicated on preserving the formulations in a state that mirrors their intended application closely, ensuring that any findings related to stability accurately reflect their performance in practical scenarios. The choice of light-resistant vials was strategic, intended to mitigate any potential degradation of the encapsulated CBD caused by light exposure, thereby maintaining the formulation’s integrity and therapeutic efficacy over time.

The colloidal stability of SLNs was assessed in various biologically relevant media, including PBS, PBS with 10% FBS, serum-free DMEM, and DMEM with 10% FBS. SLN dilutions (1:200) were prepared in each medium, and size and PDI were measured as previously described. Samples were then incubated at 37 °C for varying durations before analysis [103].

### 3.8. In Vitro Inflammation Study

#### 3.8.1. Cell Culture

SW 1353 human chondrocytes (HTB 94™; passage number 31–47, ATCC^®^, Manassas, VA, USA) and RAW 264.7 murine macrophages (TIB-71™; passage number 13–26, ATCC^®^, Manassas, VA, USA) cell lines were cultured in Dulbecco’s modified Eagle’s medium (DMEM) supplemented with 10% fetal bovine serum and 100 units/mL of penicillin/streptomycin (Gibco™ Thermo Fisher Scientific Inc., Waltham, MA, USA) under a humidified atmosphere containing 5% CO_2_ at 37 °C.

#### 3.8.2. Cytotoxicity

Seeded cells were pre-conditioned for 24 h and then treated with various concentrations of free CBD and CBD-loaded formulations (0.25 to 1.0 μg/mL) in 500 μL of serum-free DMEM media. Following 24 h of treatment, the media were replaced with a solution of MTT (Thiazolyl blue tetrazolium bromide; Sigma-Aldrich, St. Louis, MO, USA) in PBS (0.5 mg/mL), and the plates were incubated at 37 °C for 2 h. After removing the MTT-containing media, DMSO (Dimethyl sulfoxide; Sigma-Aldrich, St. Louis, MO, USA) was added to dissolve formazan crystals, and plates were kept in the dark for 10 min. Absorbance at 570 nm was measured using a microplate reader (CLARIOstar Plus, BGM Labtech Cary, NC, USA).

#### 3.8.3. Cell Stimulation and Treatment

To assess the potential anti-inflammatory activity of CBD-SLNs on RAW 264.7 and SW 1353 cells, ROS, nitrite, and cytokine expression levels were measured. Cells were cultured in 24-well plates until 80% confluency. Then, RAW 264.7 and SW 1353 cells were pretreated with LPS (100 ng/mL) and IL-1β (5 ng/mL) for 4 h, followed by treatment with either free CBD or CBD-SLNs. The optimal inflammatory induction concentration was determined to avoid cytotoxicity. After 24 h, the medium was collected, and nitrite and cytokine levels were assessed.

#### 3.8.4. Cytokine Assay

The concentrations of TNF-α and IL-6 in the culture media were quantified using ELISA kits (BioLegend, San Diego, CA, USA) following the manufacturer’s instructions. Absorbance was measured at a wavelength of 450 nm using a microplate reader. The concentrations of cytokines in the culture supernatant were determined by comparing the absorbance values of the samples to standard curves for each cytokine.

#### 3.8.5. Detection of Cellular Free Radical Generation

##### ROS Generation

ROS was quantified using the DCF-DA (Dichlorodihydrofluorescein diacetate or 2′,7′-dichlorofluorescein diacetate; Sigma-Aldrich, St. Louis, MO, USA) method, which involves the entry of DCF-DA into the cell and its reaction with reactive oxygen to form a green fluorescent compound, DCF [104]. ROS levels were estimated using a fluorometer. A 10 mM DCF-DA stock solution in methanol was diluted with serum-free DMEM to 25 μM. Cells were cultured, stimulated, and treated with CBD-SLNs following the procedure in Section 3.8.3. Afterward, cells were washed with ice-cold PBS and incubated with DCF-DA working solution for 30 min at 37 °C. A microplate reader measured fluorescence intensity at 485 nm excitation and 520 nm emission.

##### RNS Generation

RNS was quantified from the supernatant of the stimulated and treated cells, as described in Section 3.8.3. Nitrite concentration in the culture supernatant was determined using the Griess reaction. Specifically, 50 μL of culture supernatant was mixed with 50 μL of 1% (*w*/*v*) sulfanilamide (Sigma-Aldrich, St. Louis, MO, USA) in a 96-well plate. After a 5 min dark incubation, 50 μL of 2.5% (*w*/*v*) N-1-Napthylenediamine dihydrochloride (Sigma-Aldrich, St. Louis, MO, USA) was added, followed by another 5 min dark incubation. Absorbance at 520 nm was measured using a microplate reader.

### 3.9. Statistical Analysis

All data were expressed as mean ± SD. Statistical analysis was performed to determine the significant differences between means using one-way ANOVA and two-way ANOVA, followed by a post hoc test. A significance level of *p* < 0.05 was adopted for all analyses. The statistical analyses were conducted using GraphPad Prism^®^ 9.5.1 Software (San Diego, CA, USA).

## 4. Conclusions

In conclusion, our study demonstrates that CBD-SLNs offer a promising strategy for enhancing CBD’s bioavailability and therapeutic efficacy, particularly in the treatment of chronic inflammatory conditions. Employing statistical tools within the QbD approach, we optimized the formulation of CBD-SLNs, resulting in stable and uniformly dispersed nanoparticles. These CBD-SLNs demonstrated superior anti-inflammatory effects compared to free CBD, as evidenced by their significant reduction of proinflammatory cytokines and ROS in cell culture models. Incorporating CBD into SLNs effectively addresses and mitigates the limitations of free CBD’s poor water solubility and bioavailability. Our findings suggest that CBD-SLNs can significantly enhance CBD’s pharmacological profile, making it a viable candidate for further development as a therapeutic agent against chronic inflammation and possibly other conditions characterized by inflammation. Future studies should focus on the in vivo efficacy of CBD-SLNs to confirm their therapeutic potential and assess their safety profile. Additionally, exploration into this NP formulation’s long-term stability and scalability could pave the way for clinical trials and eventual commercialization. The promising results of our study contribute valuable insights into the design and application of NP delivery systems for hydrophobic compounds like CBD, with potential implications across a range of medical fields.

## Figures and Tables

**Table 1:** Experimental variables and their levels.

Variables	Levels	
	Low	High
*Independent (Factors)*		
A: GMS (g)	1.5	2.5
B: Polysorbate 80 (g)	0.6	1.0
C: Methanolic CBD (mg)	10	20
*Dependent (Responses)*		
Y_1_: Particle size (nm)	Minimized (but ≤200 nm)	
Y_2_: PDI	Minimized	
Y_3_: EE (%)	Maximized	
Y_4_: DL (%)	Maximized	

**Table 2:** BBD experimental matrix and response values for the optimization of CBD-SLNs.

No.	Factors			Responses			
	A	B	C	Y_1_	Y_2_	Y_3_	Y_4_
	(g)	(g)	(mg)	(nm)		(%)	(%)
F1	1.5	0.6	15	223 ± 13.3	0.2544	92.7 ± 0.12	1.82 ± 0.11
F2	2.5	0.6	15	300 ± 9.5	0.4440	96.5 ± 0.14	1.15 ± 0.21
F3	1.5	1.0	15	234 ± 13.0	0.4328	87.9 ± 0.12	1.75 ± 0.15
F4	2.5	1.0	15	140 ± 2.0	0.2574	90.8 ± 0.22	1.08 ± 0.12
F5	1.5	0.8	10	196 ± 4.2	0.2999	87.0 ± 0.25	1.15 ± 0.32
F6	2.5	0.8	10	183 ± 3.8	0.3165	89.4 ± 0.08	0.71 ± 0.23
F7	1.5	0.8	20	130 ± 2.2	0.2548	92.4 ± 0.04	2.45 ± 0.45
F8	2.5	0.8	20	119 ± 9.6	0.2602	94.9 ± 0.05	1.51 ± 0.32
F9	2.0	0.6	10	257 ± 8.3	0.2862	86.6 ± 2.95	0.86 ± 0.12
F10	2.0	1.0	10	127 ± 0.6	0.2770	84.6 ± 0.36	0.84 ± 0.13
F11	2.0	0.6	20	122 ± 2.3	0.2516	96.1 ± 0.13	1.91 ± 0.11
F12	2.0	1.0	20	120 ± 2.3	0.2043	91.8 ± 0.04	1.82 ± 0.43
F13 *	2.0	0.8	15	139 ± 1.8	0.2914	90.2 ± 0.17	1.34 ± 0.29
F14 *	2.0	0.8	15	138 ± 2.3	0.2905	91.9 ± 1.54	1.37 ± 1.23
F15 *	2.0	0.8	15	136 ± 2.7	0.2844	91.3 ± 1.39	1.36 ± 1.10

(*) Are the three replicated center points of the BBD; **Abbreviations:** A = GMS, B = Polysorbate 80, C = methanolic CBD, Y_1_ = particle size, Y_2_ = PDI, Y_3_ = EE, and Y_4_ = DL; data are presented as the mean ± SD (n = 3).

**Table 3:** The optimal conditions and the analysis of the predicted and observed values.

Optimal Conditions	Responses	Predicted Values	95% PI Low	Observed Values	95% PI High	% Error
A: 1.60 (g)	Y_1_	119.36	112.47	123.40 ± 2.00	126.24	3.38
B: 0.62 (g)	Y_2_	0.1910	0.1724	0.2099 ± 1.00	0.2144	9.89
C: 20 (mg)	Y_3_	95.33	94.18	95.16 ± 0.14	96.47	−0.17
	Y_4_	2.35	2.32	2.36 ± 0.05	2.38	0.43

**Abbreviations:** A = GMS, B = Polysorbate 80, C = methanolic CBD, Y_1_ = particle size (nm), Y_2_ = PDI, Y_3_ = EE (%), and Y_4_ = DL (%); data are presented as the mean ± SD (n = 6).

**Table 4:** Summary of the best-fit model for the release kinetics mechanisms of free CBD and CBD-SLNs.

Best-Fit Model	Presence of HSA	Parameter	R^2^	AIC	MSC
Free CBD					
Korsmeyer–Peppas	With	K_KP_ = 27.641 n = 0.395	0.9630	60.71	2.5318
First-order	Without	K_1_ = 0.314	0.9731	59.29	2.9942
CBD-SLNs					
Korsmeyer–Peppas	With	K_KP_ = 21.174 n = 0.410	0.9943	37.17	4.3967
Korsmeyer–Peppas	Without	K_KP_ = 23.793 n = 0.415	0.9769	54.29	3.0254

## Data Availability

Data are contained within the article.

## Supplementary Materials

The supporting information can be downloaded at: https://www.mdpi.com/article/10.3390/ijms25094744/s1.

## Abbreviations

ANOVA	Analysis of variance
BBD	Box-Behnken design
BCS	Biopharmaceutics Classification System
CBD	Cannabidiol
CYP	Cytochrome P
DCF-DA	Dichlorodihydrofluorescein diacetate or 2′,7′-dichlorofluorescein diacetate
DL	Drug loading
DMEM	Dulbecco’s Modified Eagle Medium
DMSO	Dimethyl sulfoxide
EE	Encapsulation efficiency
ELISA	Enzyme-linked Immunosorbent Assay
EMA	European Medicines Agency
FBS	Fetal bovine serum
FDA	Food and Drug Administration
FTIR	Fourier Transform Infrared Spectroscopy
GMS	Glyceryl monostearate
GRAS	Generally recognized as safe
HRMS	High-Resolution Mass Spectrometry
HSA	Human serum albumin
IL	Interleukin
JAKs	Janus kinases
LPS	Lipopolysaccharides
MMPs	Matrix metalloproteinases
MSC	Model selection criterion
NMR	Nuclear Magnetic Resonance
NO	Nitric oxide
OA	Osteoarthritis
PBS	Phosphate buffer solution
PDI	Polydispersity index
PPARs	Peroxisome proliferator-activated receptors
QbD	Quality-by-Design
RASFs	Rheumatoid arthritis synovial fibroblasts
RNS	Reactive nitrogen species
ROS	Reactive oxygen species
RSM	Response Surface Methodology
RT	Room temperature
SLN	Solid lipid nanoparticle
TNF-α	Tumor necrosis factor-alpha
XRD	X-ray diffraction
