# Supplementary material for: Cannabidiol-Loaded Solid Lipid Nanoparticles Ameliorate the Inhibition of Proinflammatory Cytokines and Free Radicals in an In Vitro Inflammation-Induced Cell Model
Source: Int J Mol Sci. 2024 Apr 26;25(9):4744. doi: 10.3390/ijms25094744 (PMC11083812; doi:10.3390/ijms25094744)
Supplement: Supplementary file 1 [file ijms-25-04744-s001.zip › ijms-2947638-supplementary.pdf]

International Journal of *Molecular Sciences*

Article

Cannabidiol-Loaded Solid Lipid Nanoparticles Ameliorate the Inhibition of Proinflammatory Cytokines and Free Radicals in an In Vitro Inflammation-Induced Cell Model

Khent Primo Alcantara, John Wilfred T. Malabanan, Nonthaneth Nalinratana, Worathat Thitikornpong, Pornchai Rojsitthisak and Pranee Rojsitthisak

Supplementary information

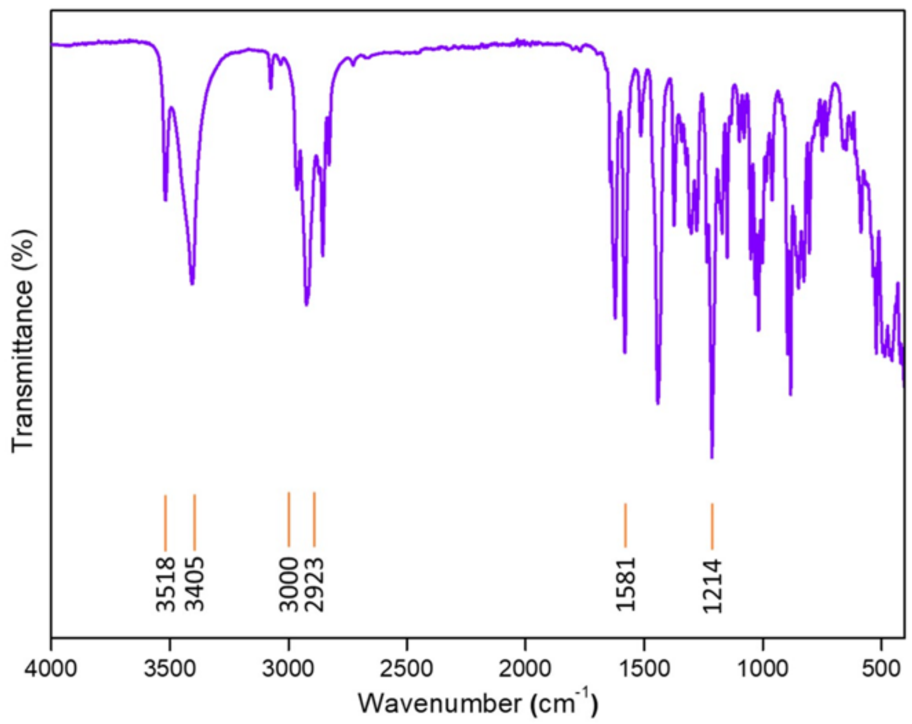

Figure S1. FT-IR spectrum of cannabidiol (CBD).

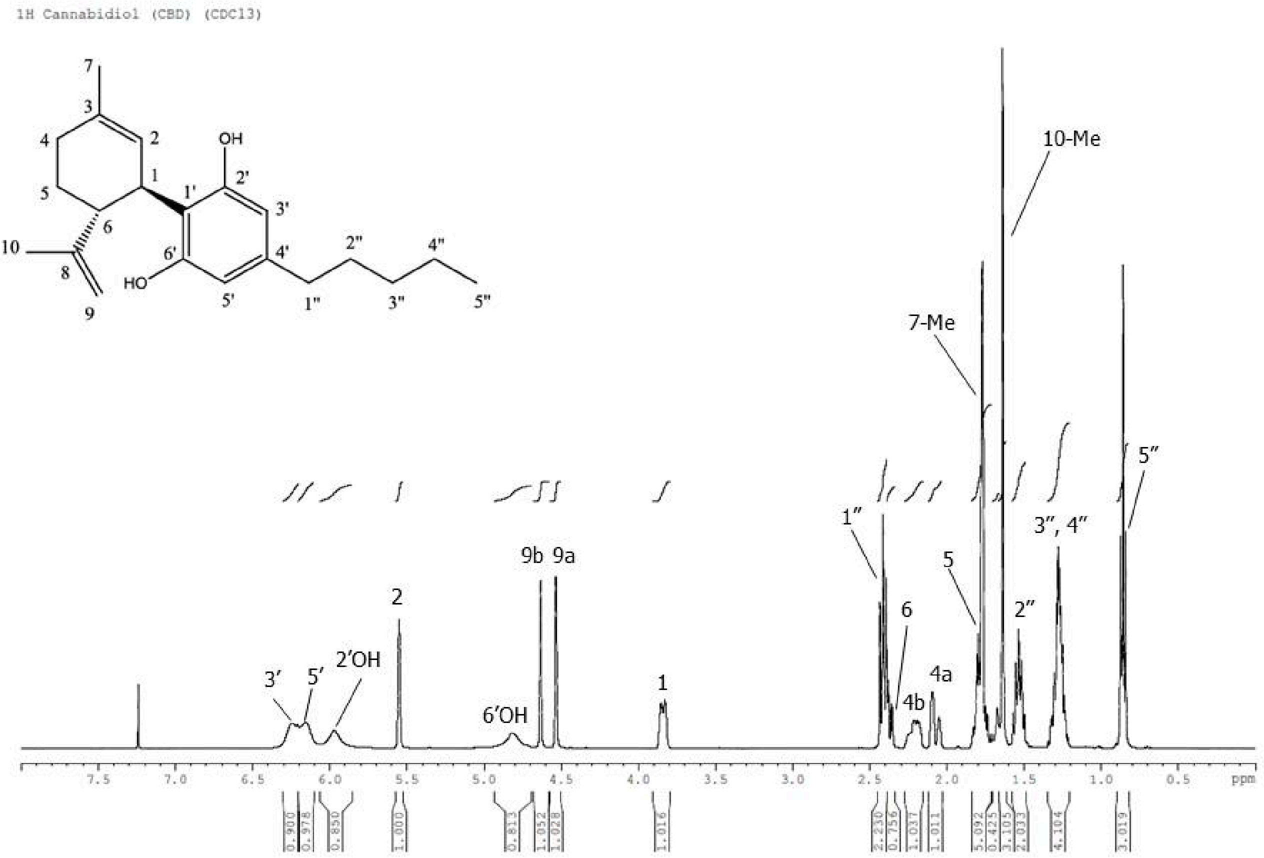

Figure S2. ^{1}H-NMR spectrum of cannabidiol (CBD).

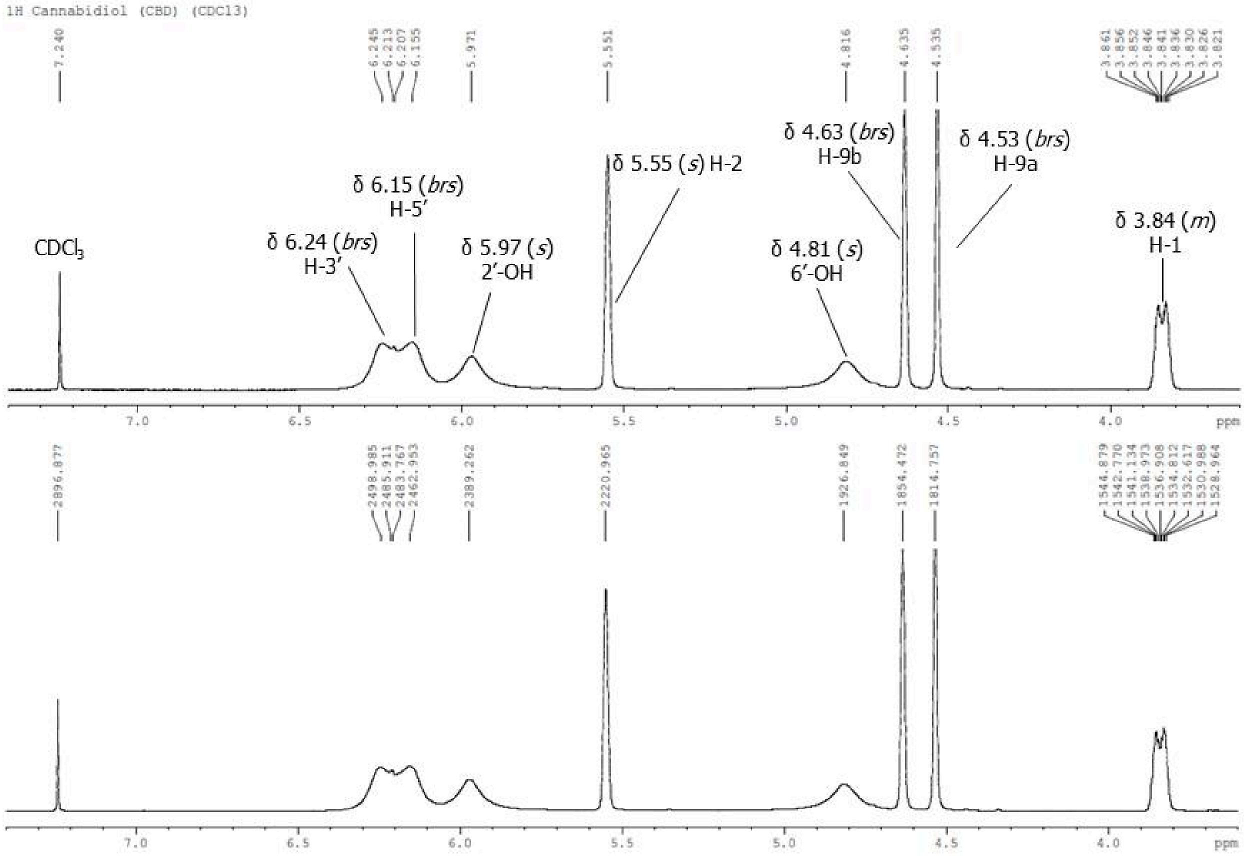

Figure S2. ^{1}H-NMR spectrum of cannabidiol (CBD) (cont.).

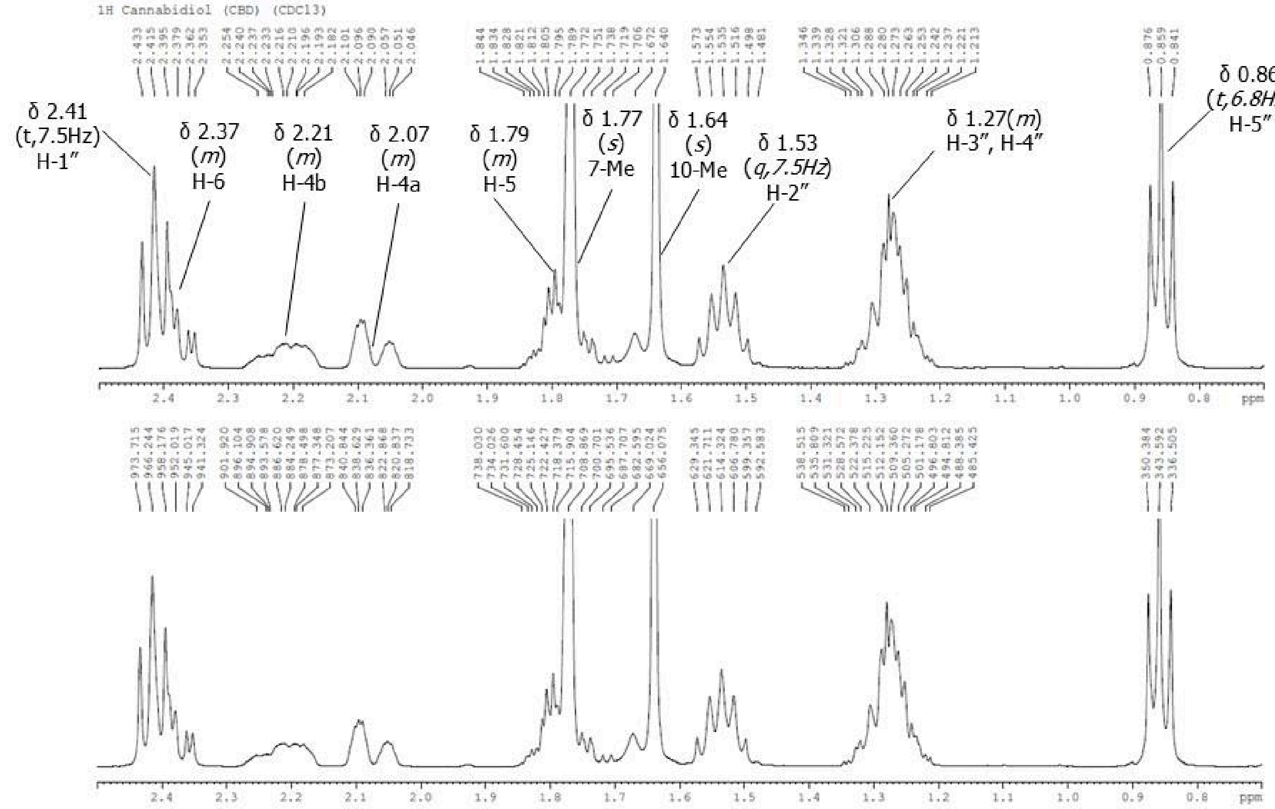

Figure S2. ^{1}H-NMR spectrum of cannabidiol (CBD) (cont.).

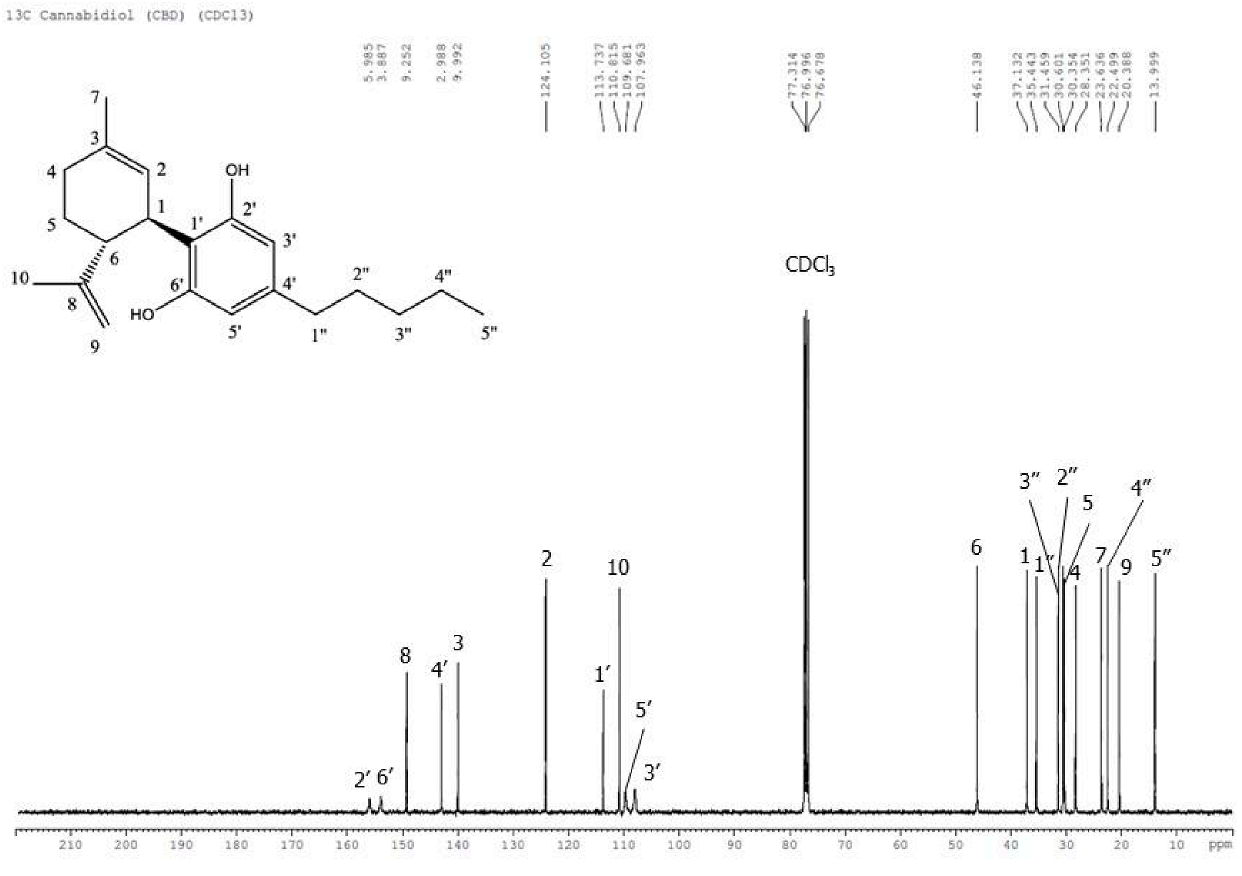

Figure S3. ^{13}C-NMR spectrum of cannabidiol (CBD).

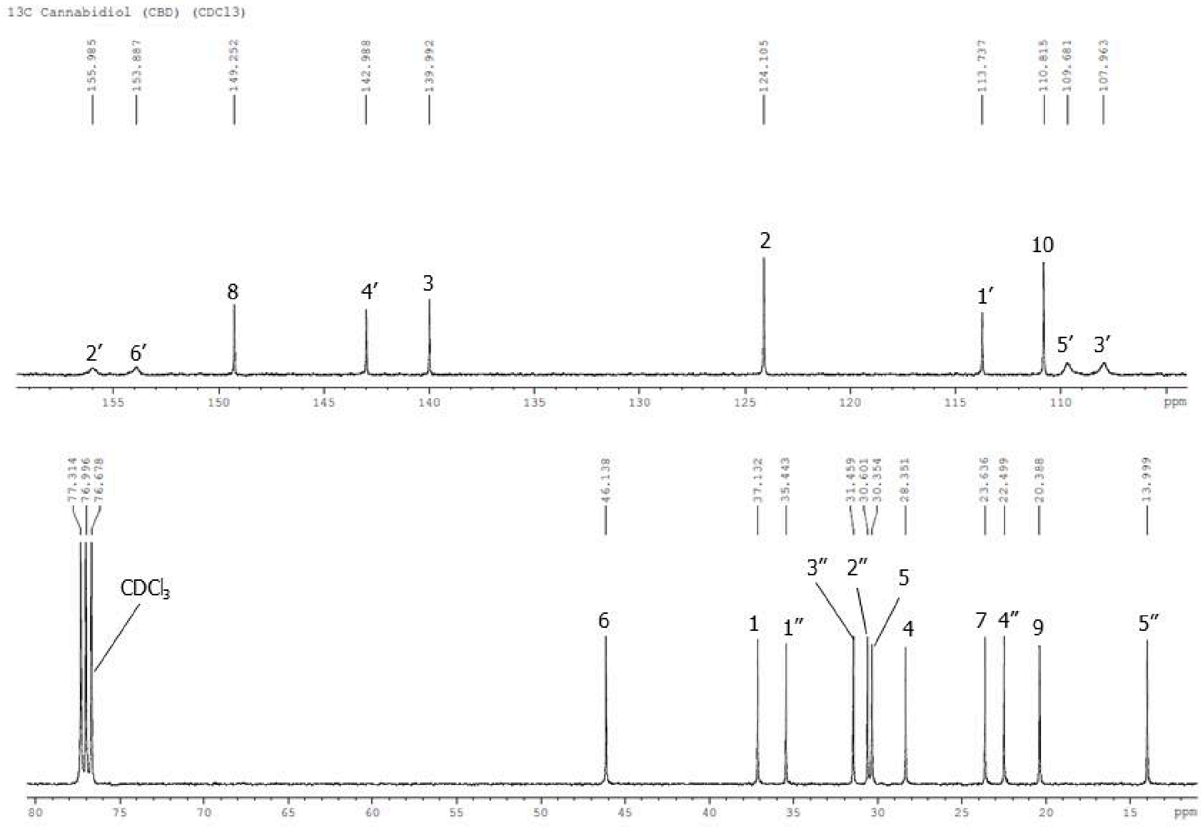

Figure S3. ^{13}C-NMR spectrum of cannabidiol (CBD) (cont.).

Acquisition Parameter

Source Type	ESI	Ion Polarity	Positive	Set Nebulizer	2.0 Bar
Focus	Not active			Set Dry Heater	200 °C
Scan Begin	50 m/z	Set Capillary	4500 V	Set Dry Gas	8.0 l/min
Scan End	2000 m/z	Set End Plate Offset	-500 V	Set Divert Valve	Waste

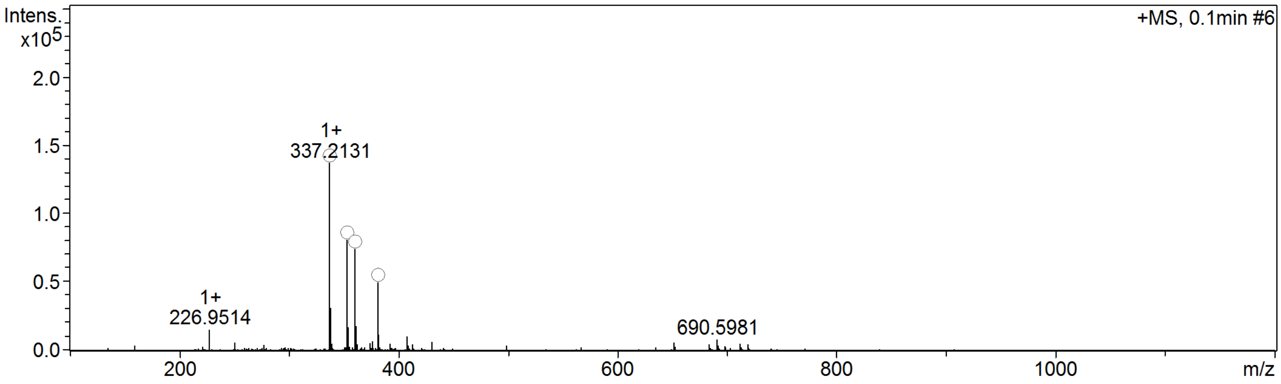

Figure S4. High-resolution mass spectrometry (HRMS) spectrum of cannabidiol (CBD).

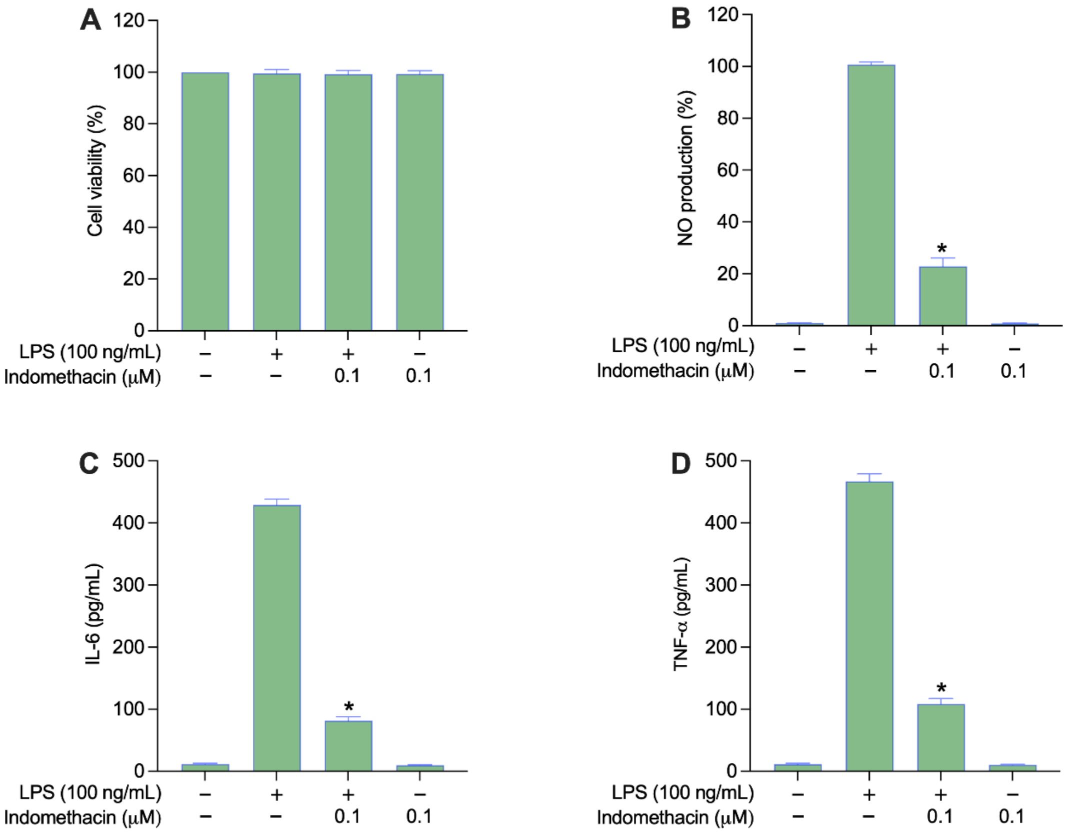

Figure S5. In vitro cell study of the positive control, Indomethacin, in RAW 264.7 murine macrophages. **(A)** Cytotoxic effect of indomethacin in LPS-stimulated RAW 264.7 murine macrophages. The effects of indomethacin on **(B)** NO production and the suppression of proinflammatory cytokines, including **(C)** IL-6 and **(D)** TNF-α, in LPS-stimulated RAW 264.7 macrophages. Indomethacin, employed as a positive control in the in vitro studies with RAW 264.7 murine macrophages, exhibited notable efficacy in reducing NO production and pro-inflammatory cytokine levels upon LPS stimulation. This affirms indomethacin as a positive control for evaluating anti-inflammatory properties, thereby validating the reliability of results. Data are presented as the mean ± SD (n=4). *$p < 0.05$ compared to LPS-stimulated cells without treatment.

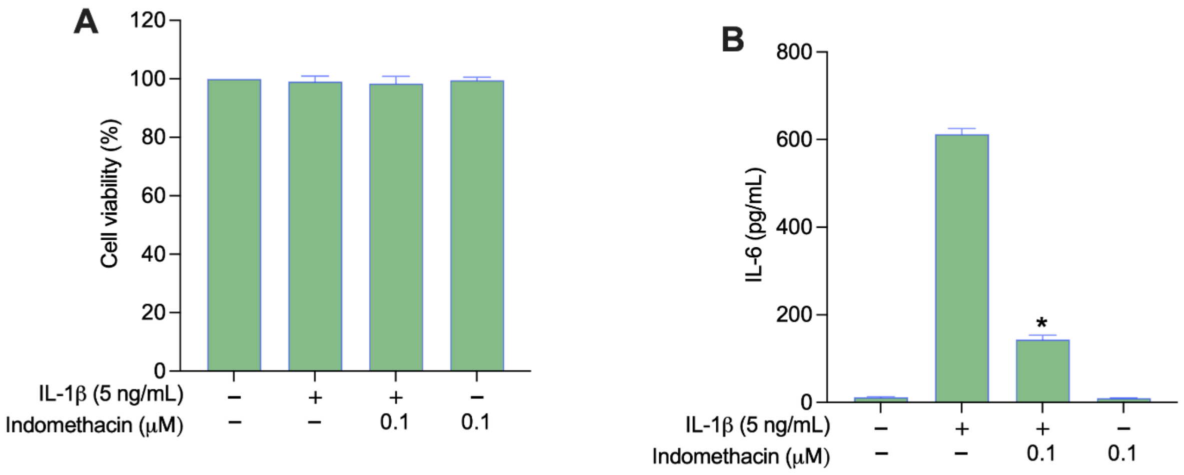

Figure S6. In vitro cell study of the positive control, Indomethacin, in SW 1353 human chondrocytes. **(A)** Cytotoxic effect of indomethacin in IL-1β-stimulated SW 1353 cells. **(B)** Effects of Indomethacin on IL-6 in IL-1β-stimulated SW 1353 cell lines. Indomethacin, employed as a positive control in the in vitro studies with SW 1353 cells, exhibited notable efficacy in reducing pro-inflammatory cytokine levels upon IL-1β stimulation. This affirms indomethacin as a positive control for evaluating anti-inflammatory properties, thereby validating the reliability of results. Data are presented as the mean ± SD (n=4). *$p < 0.05$ compared to IL-1β-stimulated cells without treatment.

Table S1. Results of ANOVA for Particle size.

Source	Sum of Squares	df	Mean Square	*F*-value	*p*-value	
Model	47087.73	8	5885.97	601.11	< 0.0001	significant
A-GMS	221.13	1	221.13	22.58	0.0032	
B-Tween 80	9912.32	1	9912.32	1012.31	< 0.0001	
C-CBD	9220.82	1	9220.82	941.69	< 0.0001	
AB	7276.94	1	7276.94	743.17	< 0.0001	
BC	4126.14	1	4126.14	421.39	< 0.0001	
A^2	7025.77	1	7025.77	717.52	< 0.0001	
B^2	6875.19	1	6875.19	702.14	< 0.0001	
C^2	2140.75	1	2140.75	218.63	< 0.0001	
Residual	58.75	6	9.79			
Lack of Fit	54.69	4	13.67	6.74	0.1334	not significant
Pure Error	4.06	2	2.03			
Cor Total	47146.48	14				

Table S2. Results of ANOVA for PDI.

Source	Sum of Squares	df	Mean Square	*F*-value	*p*-value	
Model	0.0575	7	0.0082	79.07	< 0.0001	significant
A-GMS	0.0002	1	0.0002	1.58	0.2494	
B-Tween 80	0.0005	1	0.0005	5.04	0.0596	
C-CBD	0.0054	1	0.0054	52.43	0.0002	
AB	0.0333	1	0.0333	320.76	< 0.0001	
A^2	0.0069	1	0.0069	66.45	< 0.0001	
B^2	0.0008	1	0.0008	8.17	0.0244	
C^2	0.0089	1	0.0089	85.89	< 0.0001	
Residual	0.0007	7	0.0001			
Lack of Fit	0.0007	5	0.0001	9.62	0.0968	not significant
Pure Error	0.0000	2	0.0000			
Cor Total	0.0582	14				

Table S3. Results of ANOVA for EE.

Source	Sum of Squares	df	Mean Square	*F*-value	*p*-value	
Model	142.18	3	47.39	65.97	< 0.0001	significant
A-GMS	16.70	1	16.70	23.25	0.0005	
B-Tween 80	48.66	1	48.66	67.73	< 0.0001	
C-CBD	76.82	1	76.82	106.92	< 0.0001	
Residual	7.90	11	0.7184			
Lack of Fit	6.29	9	0.6994	0.8697	0.6408	not significant
Pure Error	1.61	2	0.8041			
Cor Total	150.08	14				

Table S4. Results of ANOVA for DL.

Source	Sum of Squares	df	Mean Square	*F*-value	*p*-value	
Model	3.18	6	0.5299	2747.41	< 0.0001	significant
A-GMS	0.9385	1	0.9385	4866.04	< 0.0001	
B-Tween 80	0.0091	1	0.0091	47.25	0.0001	
C-CBD	2.13	1	2.13	11055.40	< 0.0001	
AC	0.0625	1	0.0625	324.07	< 0.0001	
BC	0.0012	1	0.0012	6.35	0.0358	
A^2	0.0358	1	0.0358	185.37	< 0.0001	
Residual	0.0015	8	0.0002			
Lack of Fit	0.0011	6	0.0002	0.7687	0.6606	not significant
Pure Error	0.0005	2	0.0002			
Cor Total	3.18	14				

Table S5. Summary of the regression analyses of the responses.

Response	Suggested Model	*p*-value, overall model	*p*-value, lack-of-fit	Adjusted R^2	Predicted R^2	Adeq precision
Particle size	Quadratic	< 0.0001	0.1022	0.9965	0.9813	73.8627
PDI	Quadratic	< 0.0001	0.1279	0.9840	0.9154	30.9626
EE	Linear	< 0.0001	0.6408	0.9330	0.8979	25.4282
DL	Quadratic	0.0006	0.4438	0.9987	0.9948	181.0404

Table S6. Release kinetic modeling of CBD-SLN.

Model	Presence of HSA	Parameter	R^2	AIC	MSC
Zero-order ($F = k_0 \cdot t$)	✓	$K_0 = 4.256$	0.4317	81.19	-0.0050
	✗	$K_0 = 4.832$	0.4272	84.40	0.0155
First-order ($F = 100 \cdot e^{-k_1 t}$)	✓	$K_1 = 0.088$	0.8669	66.67	1.4468
	✗	$K_1 = 0.116$	0.9243	64.16	2.0390
Higuchi ($F = k_H \cdot t^{0.5}$)	✓	$K_H = 17.069$	0.9734	50.57	3.0568
	✗	$K_H = 19.434$	0.9586	58.1162	2.6437
Korsmeyer-Peppas ($F = k_{KP} \cdot t^n$)	✓	$K_{KP} = 21.174$, $n = 0.410$	0.9943	37.17	4.3967
	✗	$K_{KP} = 23.793$, $n = 0.415$	0.9769	54.2933	3.0254
Hixson-Crowell ($F = 100 \cdot [1 - (1 - k_{HC} \cdot t)^3]$)	✓	$K_{HC} = 0.025$	0.8013	70.68	1.0458
	✗	$K_{HC} = 0.033$	0.8816	68.64	1.5916

Table S7. Release kinetic modeling of Free CBD.

Model	Presence of HSA	Parameter	R^2	AIC	MSC
Zero-order ($F = k_0 \cdot t$)	☑	$K_0 = 5.313$	0.3400	87.51	-0.1486
	☒	$K_0 = 6.441$	-0.0347	95.80	-0.6568
First-order ($F = 100 \cdot e^{-k_1 t}$)	☑	$K_1 = 0.146$	0.9346	64.39	2.1629
	☒	$K_1 = 0.314$	0.9731	59.29	2.9942
Higuchi ($F = k_H \cdot t^{0.5}$)	☑	$K_H = 21.506$	0.9332	64.60	2.1420
	☒	$K_H = 26.879$	0.8142	78.62	1.0603
Korsmeyer-Peppas ($F = k_{KP} \cdot t^n$)	☑	$K_{KP} = 27.641$<br>$n = 0.395$	0.9630	60.71	2.5318
	☒	$K_{KP} = 40.982$<br>$n = 0.322$	0.9229	71.82	1.7404
Hixson-Crowell ($F = 100 \cdot [1 - (1 - k_{HC} \cdot t)^3]$)	☑	$K_{HC} = 0.040$	0.9076	67.85	1.8178
	☒	$K_{HC} = 0.062$	0.8964	72.78	1.6448
